# Pear Peel–Enriched Residue Extract Attenuates Ethanol‐Induced Liver Injury Through Regulation of Antioxidant Signaling and Lipid Metabolism

**DOI:** 10.1155/adpp/6147577

**Published:** 2026-11-04

**Authors:** Laxmi Sen Thakuri, Ducdat Le, Narayan Sah Sonar, Hye Bin Park, Laxman Subedi, Ki-Sun Lee, Jeong-Ho Shon, Jin Woo Park, Mina Lee, Dong Young Rhyu

**Affiliations:** ^1^ School of Food and Pharmaceutical Engineering Mokpo National University Mokpo 58554 Jeonnam South Korea mokpo.ac.kr; ^2^ Bio-Medicine Advanced Formulation Research Center Regional Leading Research Center Mokpo National University Mokpo 58554 Jeonnam South Korea mokpo.ac.kr; ^3^ College of Pharmacy and Research Institute of Life and Pharmaceutical Sciences Sunchon National University Suncheon Jeonnam-Gwangju 57922 South Korea sunchon.ac.kr; ^4^ Department of Natural Cosmetics Science and AI Digital Bioscience Research Institute Sunchon National University Suncheon Jeonnam-Gwangju 57922 South Korea sunchon.ac.kr; ^5^ AI Bio Autonomous Research Core Facility Center Sunchon National University Suncheon Jeonnam-Gwangju 57922 South Korea sunchon.ac.kr; ^6^ Department of Biomedicine, Health & Life Convergence Sciences BK21 Four Mokpo National University Mokpo 58554 Jeonnam South Korea mokpo.ac.kr; ^7^ Department of Pharmacy Mokpo National University Mokpo 58554 Jeonnam South Korea mokpo.ac.kr; ^8^ Good F&B Naju 58245 South Korea

**Keywords:** ethanol-induced liver injury, molecular docking, oxidative stress, pear peel–enriched residue extract (PPE)

## Abstract

Excessive alcohol consumption induces progressive liver injury through oxidative stress, lipid accumulation, and inflammation, primarily driven by dysregulated ethanol (EtOH) metabolism. Given the growing interest in plant‐derived phytochemicals for hepatoprotection, pear (*Pyrus pyrifolia*) has attracted attention due to its antioxidant and lipid‐regulating properties; however, the bioactive fractions and mechanisms underlying the hepatoprotective effects of pear peel–enriched residue extracts (PPE) remain unclear. Therefore, we evaluated the hepatoprotective potential of PPE against EtOH‐induced liver injury using in vitro, in vivo, and in silico approaches. In HepG2 cells, PPE reduced oxidative stress, restored cell viability, and reduced intracellular EtOH and acetaldehyde levels more effectively than pear extract, with efficacy comparable to silymarin. In EtOH‐treated C57BL/6 mice, PPE attenuated liver injury markers, reduced hepatic lipid accumulation, and improved histopathological alterations. PPE also modulated the expression of proteins associated with oxidative stress and lipid metabolism. GNPS–FBMN analysis putatively annotated over 120 phytochemicals (or metabolites), and in silico analyses suggested potential interactions between selected metabolites and proteins involved in EtOH metabolism and lipid regulation. These findings suggest that PPE shows hepatoprotective potential and warrants further investigation as a potential candidate for the mitigation of alcohol‐associated liver disease.

## 1. Introduction

Alcohol‐associated liver disease (ALD) is a major clinical and public health concern encompassing a progressive spectrum of hepatic disorders resulting from chronic ethanol (EtOH) consumption [1]. The pathological continuum of ALD ranges from simple steatosis and steatohepatitis to fibrosis, cirrhosis, and hepatocellular carcinoma [1, 2]. Excessive alcohol consumption contributes substantially to the global disease burden, accounting for approximately 5% of worldwide morbidity and mortality [3]. Although the development and severity of ALD are closely correlated with the duration and quantity of alcohol consumption, they are also modulated by genetic predispositions and metabolic factors [4]. Upon ingestion, EtOH is primarily metabolized in the liver by alcohol dehydrogenase (ADH), producing acetaldehyde, a highly reactive and hepatotoxic intermediate. Acetaldehyde is subsequently converted to acetate by aldehyde dehydrogenase (ALDH) [5]. However, under chronic or excessive EtOH exposure, the microsomal EtOH‐oxidizing system, especially cytochrome P450 2E1 (CYP2E1), is upregulated [5, 6]. This metabolic shift amplifies the generation of reactive oxygen species (ROS), resulting in oxidative stress, which initiates and perpetuates hepatocellular injury [7]. This redox imbalance contributes to several downstream pathological processes central to ALD progression, including mitochondrial dysfunction, lipid peroxidation, endoplasmic reticulum (ER) stress, and the activation of proinflammatory signaling pathways [8]. In addition, ROS promote the upregulation of lipogenic transcription factors such as sterol regulatory element‐binding protein‐1c (SREBP‐1c) and fatty acid synthase (FAS), which contribute to hepatic triglyceride (TG) accumulation [9, 10]. With advancement of liver damage, hepatic stellate cell activation and extracellular matrix (ECM) deposition drive fibrogenesis, ultimately leading to irreversible structural damage and compromised liver function [11]. Although oxidative stress contributes importantly to hepatic lipid dysregulation, alcohol‐induced steatosis results from multiple interconnected processes involving altered lipid synthesis, oxidation, transport, inflammatory signaling, and cellular stress responses [10, 11].

Given the multifactorial nature of ALD and the lack of approved pharmacological treatments, interest in natural compounds capable of targeting multiple pathogenic pathways has been growing [12]. Phytochemicals with antioxidant, anti‐inflammatory, and lipid‐modulating properties hold particular promise for mitigating EtOH‐induced hepatic injury [13, 14]. In parallel, herbal products remain the primary healthcare resources for approximately 75% of the global population and, together with conventional medicine, form major pillars of global healthcare systems [15]. These observations highlight the continued importance of plant‐derived bioactive compounds as potential therapeutic agents for metabolic and liver diseases. Among them, extracts from *Pyrus pyrifolia* have attracted attention due to their diverse phytochemical composition and potential biological activities [16]. Previous studies have suggested that pear‐derived extracts enhance ADH and ALDH activities, promoting more efficient detoxification of acetaldehyde [17]. Consistent with these observations, both in vitro and in vivo models of alcohol‐induced liver injury have demonstrated that these extracts attenuate oxidative damage, restore antioxidant capacity, and suppress hepatic lipid peroxidation [18]. Notably, the distribution of phytochemicals within pear tissues is not uniform. Pear peel tissues are particularly enriched in polyphenols (e.g., chlorogenic acid and arbutin), flavonoids, and triterpenoids that exhibit ROS‐scavenging activity and support antioxidant defense systems [17, 18]. During industrial or household processing of pears, juice extraction generates a solid residue that remains enriched in peel‐associated phytochemicals and other insoluble fruit components. Utilization of this residue may therefore offer a value‐added approach for recovering potentially bioactive constituents from an otherwise underutilized by‐product. However, the phytochemical characteristics and hepatoprotective potential of this peel‐enriched residue remain insufficiently characterized relative to conventional pear‐derived extracts, leaving a clear knowledge gap regarding its comparative efficacy and mechanism of action against EtOH‐induced liver injury. Therefore, the present study aimed to systematically investigate the hepatoprotective effects of pear peel–enriched residue extract (PPE), prepared from peel‐rich residue remaining after juice extraction, against EtOH‐induced liver injury using cellular, animal, and molecular approaches. By integrating phytochemical profiling with mechanistic protein expression analyses and in silico docking of active compounds, this study aimed to investigate the potential mechanism of PPE, with particular emphasis on oxidative stress, lipid metabolism, and molecular changes associated with EtOH‐induced liver injury.

## 2. Materials and Methods

### 2.1. Reagents

2՛,7՛‐Dichlorofluorescein diacetate (DCFH‐DA), dimethyl sulfoxide (DMSO), ethyl alcohol (E7023), and 3‐(4,5‐dimethylthiazol‐2‐yl)‐2,5‐diphenyl tetrazolium bromide (MTT) were purchased from Sigma‐Aldrich (St. Louis, MO, USA). Primary antibodies *β*‐actin (sc‐47778), collagen type 1 alpha 1 (COL1A1; sc‐293182), FAS (sc‐48357), and SREBP‐1c (sc‐365513) were purchased from Santa Cruz Biotechnology (Dallas, TX, USA). Other primary antibodies, such as glutamate–cysteine ligase catalytic subunit (GCLC; #48005), NRF2 (#12721), NF‐κB (#8242), antimouse IgG (#7076), and antirabbit IgG (#7074), were purchased from Cell Signaling Technology (Danvers, MA, USA). CYP2E1 (OTI5F11), DAPI and Hoechst nucleic acid stain (#D1306), Folin–Ciocalteu reagent, 2,2‐diphenyl‐1‐picrylhydrazyl (DPPH), and chemiluminescent substrates were obtained from Thermo Fisher Scientific (Waltham, MA, USA). Protein assay dye, acrylamide/bis solution, polyvinylidene difluoride (PVDF) membranes, and all reagents used for western blotting were purchased from Bio‐Rad Laboratories (Hercules, CA, USA). All solvents and reagents were of analytical grade and purchased from Sigma‐Aldrich, unless otherwise specified.

### 2.2. Extract Preparation

Fresh whole pear fruit (*P. pyrifolia*, 1 kg) was mixed with distilled water (w/v) and heated at 60°C for 4 h with continuous stirring. The extract was filtered to remove insoluble residues, and the filtrate was concentrated under reduced pressure at 60°C until it reached 72°Brix. The resulting concentrated whole pear extract (PE) contained a final moisture content of 9.28% and was stored at 4°C. The solid residue remaining after juice extraction, which was enriched in peel tissues and other insoluble fruit components, was used for preparation of the PPE. This residue (approximately 1 kg) was mixed with distilled water (w/v) and extracted at 85°C for 2 h. Following extraction, the solution was filtered and concentrated under reduced pressure at 48°C until it reached 48°Brix. The concentrated PPE had a final moisture content of 9.11% and was stored at 4°C for subsequent experiments. Because PPE was obtained from the remaining residue after juice extraction, it represents an extract enriched in peel‐associated and insoluble fruit fraction relative to the juice‐derived extract (PE).

### 2.3. Total Phenol Content and DPPH Scavenging Activity

Total phenolic content was determined using the Folin–Ciocalteu method with slight modification. Briefly, 200 µL of extract solution prepared from weighed PE and PPE (1 mg/mL) was mixed with 1.4 mL of distilled water and 100 µL of Folin–Ciocalteu reagent. After incubation for 30 s at room temperature, 300 µL of 20% sodium carbonate solution was added to the reaction mixture. The mixture was then incubated at room temperature for 2 h in the dark. Finally, absorbance was measured at 765 nm. Total phenolic content was calculated using a gallic acid standard curve and expressed as mg gallic acid/100 g of sample. The radical scavenging activity of PE and PPE was evaluated using the DPPH assay. Briefly, 100 µL of PE and PPE (50, 250, 500 µg/mL) were added with 100 µL of 150 µM DPPH solution, followed by incubation at room temperature for 30 min in the dark. After incubation, absorbance was measured at 540 nm, and inhibition rate was calculated as follows: Inhibition rate (%) = (1 − [A_Sample − A_Sample blank]/[A_Control − A_Control blank])^∗^ 100.

### 2.4. Cell Culture and Cytotoxicity Assessment

HepG2 cells (ATCC, Manassas, VA, USA) were cultured in Dulbecco’s Modified Eagle Medium supplemented with 10% fetal bovine serum (FBS) and 1% penicillin–streptomycin under 5% CO_2_ at 37°C. For cytotoxicity evaluation, cells were treated with PE and PPE at varying concentrations (0.05 to 0.4°Brix) for 24 h. Cell viability was assessed using the MTT assay, with MTT (1 mg/mL) added to the cells and incubated for 3 h. After solubilizing the formazan crystals, absorbance was determined at 540 nm, and the resulting values were normalized to those of untreated control cells to express cell viability.

### 2.5. EtOH‐Induced Hepatotoxicity Model and PPE Treatment

HepG2 cells were first exposed to 3% EtOH for 24 h. Following this exposure, cells were treated with various concentrations of PE and PPE (0.05–0.4°Brix), with or without EtOH, for a further 24 h. Silymarin (Sm; 100 µM) was used as a reference hepatoprotective agent.

### 2.6. Measurement of Intracellular ROS

Intracellular ROS levels were determined using 10 µM DCFH‐DA. Following treatment, HepG2 cells were incubated with DCFH‐DA for 1 h, washed with phosphate‐buffered saline (PBS), followed by Hoechst DAPI staining (1 µM) for an additional 5 min. The stained cells were washed with PBS, and fluorescence was measured using a Varioskan LUX reader (485/527 nm; Thermo Fisher Scientific). Images of ROS were captured using a CKX53 fluorescence microscope (Olympus, Tokyo, Japan).

### 2.7. Animal Husbandry and Experimental Design

Male C57BL/6N mice (6 weeks old) purchased from Orient Bio Inc. (Seongnam, South Korea) were maintained under controlled conditions (25 ± 2°C, 12/12°h light/dark cycle) with free access to standard chow and water. After a 1‐week acclimatization period, the animals were divided into six groups (*n* = 7 each): control (saline), EtOH (40% EtOH, 5 g/kg BW), PPE 100 mg/kg + 40% EtOH, PPE 200 mg/kg + 40% EtOH, PPE 400 mg/kg + 40% EtOH, and Sm 100 mg/kg + 40% EtOH (positive control). Each group received its assigned daily treatment by oral gavage over the 3‐week study. Body weight and food and water intake were monitored every 3 days. Following an overnight fasting period, mice were sacrificed via cervical dislocation, after which blood and liver samples were collected for subsequent analyses. All experimental procedures were reviewed and approved by the Institutional Animal Care and Use Committee of Mokpo National University (Approval No. MNU‐IACUC‐2024‐008).

### 2.8. Histological and Lipid Staining

Liver samples were fixed in 10% neutral‐buffered formalin for 48 h at room temperature, embedded in paraffin wax. Paraffin‐embedded tissues were sectioned at 4‐µm thickness using a rotary microtome and mounted on glass slides. Sections were stained with hematoxylin and eosin (H&E) to evaluate general liver histopathology and morphological changes, including hepatocyte structure, tissue architecture, and others. For Oil Red O staining, tissues were cryo‐embedded in OCT after 30% sucrose immersion, sliced (2–3 mm), stained with 0.5% Oil Red O, and nuclei were counterstained with hematoxylin. Stained sections were examined under a light microscope, and representative images were captured at 400x magnification for histological and lipid accumulation assessment.

### 2.9. Biochemical Assays

EtOH and acetaldehyde concentrations in mouse serum and tissues, as well as intracellular levels in HepG2 cells, were measured using Megazyme diagnostic assay kits (Megazyme, Bray, Ireland) according to the provided protocol by the manufacturer. Concentrations were calculated from the standard curve and initially expressed in absolute units (mg/dL for EtOH and mg/L for acetaldehyde). For graphical comparison, values were subsequently normalized relative to the control group. For tissue samples, acetaldehyde levels were further normalized to protein content and expressed as µg acetaldehyde per 100 µg protein. Serum levels of glutamate pyruvate transaminase (GPT), glutamate oxaloacetate transaminase (GOT), and TG were determined using commercial kits from Asan Pharmaceutical (Cheonggyecheon, South Korea). All assays were conducted according to the provided instructions of the manufacturer.

### 2.10. Western Blot Analysis

Total protein was extracted from liver tissues (∼100 mg) using RIPA buffer containing phosphatase and protease inhibitors. An equal amount of proteins (30 µg) from each sample was separated with 5X SDS sample loading buffer, denatured by heating at 95°C for 5 min, and separated by 8%–10% SDS–PAGE, then transferred onto PVDF membranes using a semidry transfer system. Membranes were blocked for 1 h at room temperature with 5% bovine serum albumin (BSA) in Tris‐buffered saline containing 0.1% Tween‐20 (TBST) buffer. Following blocking, membranes were incubated overnight at 4°C with primary antibodies diluted in 5% BSA/TBST, targeting the following proteins: SREBP‐1c (1:250), FAS (1:250), GCLC (1:1000), CYP2E1 (1:1000), NRF2 (1:1000), Col1A1 (1:1000), and *β*‐actin (1:2000). After primary antibody incubation, membranes were washed three times with TBST (10 min each), then incubated with horseradish peroxidase (HRP)‐conjugated secondary antibodies (1:1000) for 90 min at room temperature. Membranes were subsequently washed three additional times with TBST, and protein bands were visualized by enhanced chemiluminescence detection using an iBright CL750 system (Thermo Fisher Scientific). Band intensities were quantified by densitometric analysis using ImageJ software, and protein expression levels were normalized to *β*‐actin.

### 2.11. LC–MS/MS Conditions

Samples were filtered by using a polytetrafluoroethylene membrane (size of 0.22 μm, manufactured by Agilent Technologies, Santa Clara, CA, USA). Then, the LC–MS/MS analysis was performed following previously reported procedures [19, 20] with some modifications. The mobile phase was utilized by using a binary solvent system, comprising 0.1% formic acid buffered into both channels A (high‐grade purity water) and B (acetonitrile). The elution condition was established as detailed below: Initiate with 35%–45% B for 17 min, then increase to 100% B from 17 to 23 min, sustain 100% B for an additional 3 min, and finally revert to 35% B within 1 min for re‐equilibration. A 4 μL was injected for analysis with a consistent flow rate of 0.2 mL/min maintained during the entire analysis process.

### 2.12. GNPS–FBMN and Compound Annotation

We used the ProteoWizard MS Converter (https://proteowizard.sourceforge.io/) to transfer the raw UHPLC–MS/MS data to mzXML format, enabling subsequent spectral processing and database comparison. The converted MS/MS data were then processed to extract and align spectral features for compound annotation. Molecular networking parameters were fine‐tuned to group tandem MS spectra based on how similar they are. The resulting network had nodes that represented consensus MS^2^ spectra. Connections (edges) were made based on cosine similarity scores, which showed how closely nodes’ spectra were related. The Global Natural Products Social Molecular Networking (GNPS) platform (https://gnps.ucsd.edu) was used to create feature–based molecular networking (FBMN) illustrating chemical interactions between metabolites found in PPE. Cytoscape 3.9.0 (https://cytoscape.org/) was utilized to visualize the network. Furthermore, we used MS‐Dial to analyze and determine predicted compounds by comparing their mass data with those from public databases (GNPS, HMDB, and MassBank) [20].

### 2.13. Molecular Docking

In silico molecular docking was carried out to evaluate the interactions between selected PPE metabolites and target proteins associated with liver injury. The crystal structures of ADH (PDB ID: 1HDZ), FAS (PDB ID: 1XKT), liver fibrosis‐associated protein (PDB ID: 3GLU), and ALDH (PDB ID: 4H80) were retrieved from the Protein Data Bank. Protein and ligand preparations were conducted by using MGL tools, following our previous reports [19–22]. The docking score function was calculated by using Autodock 4.2.6. Interactions between ligand and binding sites were visualized by using the Discovery Studio 2021 program.

### 2.14. Molecular Dynamics (MD) Simulation

MD simulations were performed for the docked complexes of Compounds 26 and 119 with three target proteins, including human ADH 1B, β2 isoform (ADH; PDB; 1HDZ), the thioesterase domain of human FAS (PDB; 1XKT), and the catalytic domain of human sirtuin (SIRT31; PDB: 3GLU). MD simulations were carried out to assess the dynamic stability of protein–ligand complexes by evaluating the trajectory parameters. The experimental procedures were conducted by using GROMACS 2023.3, following our previous report [23]. Briefly, the AmberGS force field was chosen during the topology preparation and solvated in the TIP3P water model, which was within a cubic simulation box, maintaining a 1 nm distance between the complex and the box edge. Energy minimization was performed using the steepest descent minimization algorithm with a maximum of 50,000 steps and a force below 10.0 kJ/mol, followed by NVT (number of particles, volume, and temperature) and NPT (number of particles, pressure, and temperature) equilibration at 300 K and 1 bar utilizing a V‐rescale thermostat and Parrinello–Rahman barostat. After that, production MD simulations were conducted for 20,000 picoseconds (ps) for subsequent analysis. Trajectory analysis further computed the RMSD, RMSF, SASA, hydrogen bonds, and Rg values.

### 2.15. Absorption, Distribution, Metabolism, Elimination, and Toxicity (ADMET) Evaluation

The ADMET profiles of P. pyrifolia components were investigated by utilizing the admetSAR 3.0 webserver (https://lmmd.ecust.edu.cn/admetsar3/index.php; accessed on March 19, 2026) through multiple criteria, including human intestinal absorption (HIA), Caco‐2 permeability, plasma protein binding (PPB), and bioavailability profiles. The toxicological criteria assessed by selecting criteria of drug‐induced liver injury (DILI) and *Salmonella*/microsomes (AMES) scores.

### 2.16. Statistical Analysis

All experiments were conducted in biological triplicate or with *n* = 7 animals per group, unless otherwise stated. Data from in vitro experiments are presented as mean ± standard error of the mean (SEM), whereas data from in vivo experiments are presented as mean ± standard deviation (SD). Statistical comparisons were performed using one‐way ANOVA followed by Dunnett’s post hoc test in GraphPad Prism 8.0.2 (GraphPad Software, La Jolla, CA, USA). Differences were considered statistically significant at *p* < 0.05.

## 3. Results

### 3.1. Comparative Antioxidant Profiling of PE and PPE

To quantitatively differentiate the chemical properties of the two pear‐derived extracts, PE, obtained from the whole fruit, and PPE, prepared from the peel‐rich residue remaining after juice extraction, the total phenolic content of both extracts was determined using the Folin–Ciocalteu method. As shown in Supporting Figure S1A, PPE exhibited substantially higher phenolic content than PE, reaching 381.46 mg GAE/100 g extract, whereas PE contained 156.22 mg GAE/100 g extract. This corresponds to an approximately 2.4‐fold enrichment of phenolic compounds in the PPE relative to the PE.

Consistent with the phenolic enrichment, PPE demonstrated stronger radical‐scavenging activity than PE across all tested concentrations in the DPPH assay (Supporting Figure S1B). While both extracts showed concentration‐dependent increases in scavenging activity, PPE consistently produced higher inhibition rates than PE at equivalent concentrations. At 500 µg/mL, PPE approached the scavenging efficiency of the reference antioxidant ascorbic acid. Together, these findings indicate that PPE contains a higher abundance of antioxidant‐associated constituents compared with PE.

### 3.2. PE and PPE Attenuate EtOH‐Induced Oxidative Stress and Hepatotoxicity in HepG2 Cells

Following the antioxidant characterization of the extracts (Supporting Figure S1), which demonstrated substantially higher phenolic enrichment and radical‐scavenging activity in PPE compared with PE, we next evaluated whether these extracts could protect hepatocytes against EtOH‐induced oxidative injury.

HepG2 cells were treated with PE or PPE at a concentration of 0.05–0.4°Brix to assess extract‐induced cytotoxicity. As shown in Figure 1A, no significant cytotoxicity was observed in HepG2 cells treated with either PE or PPE. Treatment with 3% EtOH significantly reduced cell viability compared with the untreated control (*p* < 0.001). However, treatment with PE and PPE (0.05–0.1°Brix) significantly attenuated the EtOH‐induced cytotoxicity (Figure 1B). Furthermore, PE and PPE significantly reduced intracellular accumulation of EtOH and acetaldehyde, which were both increased by EtOH exposure. EtOH treatment also led to a marked increase in intracellular ROS levels (*p* < 0.001), whereas PE and PPE treatments significantly attenuated this increase (*p* < 0.001). These results indicate that both PE and PPE exerted protective effects against EtOH‐induced hepatotoxicity by restoring cell viability, reducing EtOH and acetaldehyde accumulation, and reducing oxidative stress. Notably, PPE consistently produced stronger cytoprotective and ROS‐suppressive effects than PE across the tested concentrations, with activity comparable to that of the reference hepatoprotective compound silymarin. Considering the high phenolic enrichment and antioxidant capacity observed for PPE, together with its superior protective efficacy in vitro, PPE was selected for subsequent in vivo and mechanistic investigations.

FIGURE 1Effects of pear extract (PE) and pear peel–enriched residue extract (PPE) extracts against ethanol‐induced hepatotoxicity. (A–B) Cell viability of HepG2 cells treated with PE or PPE with or without ethanol (EtOH, 3%) for 24 h. (C–D) EtOH and acetaldehyde levels. (E) Intracellular reactive oxygen species (ROS) detected by DCFH‐DA staining (green) and nuclear counterstaining with Hoechst (blue). (F) Fluorescence intensity of ROS production. Silymarin (Sm) was used as a positive control. Scale bar: 200 µm. Data are expressed as mean ± SEM (*n* = 3–6). ^∗^ *p* < 0.05, ^∗∗^ *p* < 0.01, ^∗∗∗^ *p* < 0.001 versus control; ^#^ *p* < 0.05, ^##^ *p* < 0.01, ^###^ *p* < 0.001 versus EtOH‐treated group.

**Figure:**
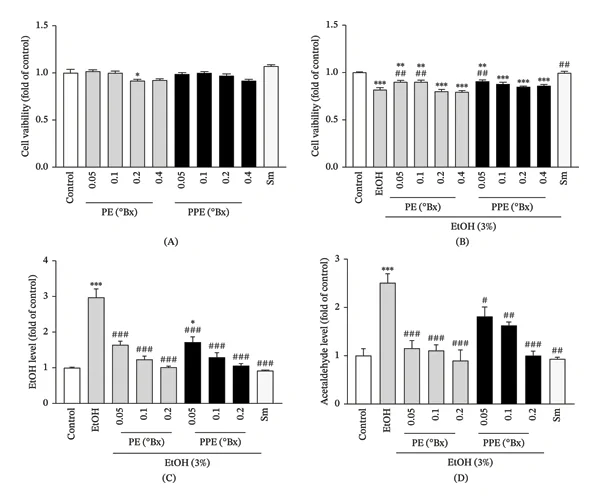

**Figure:**
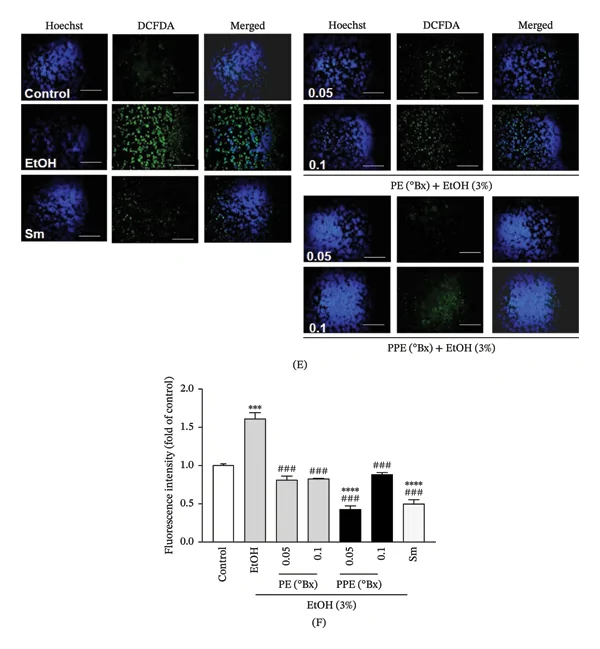

### 3.3. PPE Attenuates EtOH‐Induced Hepatic Injury and Reduces EtOH, Acetaldehyde, and Lipid Accumulation in Mice

To evaluate the protective effect of PPE, mice were administered EtOH (5 g/kg BW) with or without PPE supplementation. As shown in Figure 2A–C, EtOH administration did not significantly affect body weight or food intake in mice; however, a significant increase in water intake was observed after EtOH exposure. Importantly, EtOH treatment markedly elevated the serum levels of hepatic injury markers, including GOT and GPT, compared with those in the control group (*p* < 0.001). PPE co‐administration significantly reduced both GOT and GPT levels (*p* < 0.001 vs. EtOH), indicating a hepatoprotective effect (Figure 2D,E). Similarly, EtOH exposure significantly increased serum TG levels compared with untreated controls (*p* < 0.001) whereas PPE treatment significantly reduced this increase (Figure 2F). To further evaluate EtOH metabolism, serum EtOH and acetaldehyde concentrations were measured. EtOH administration significantly increased serum EtOH levels (Figure 2G) and acetaldehyde (Figure 2H) relative to controls. PPE supplementation significantly reduced both metabolites compared with the EtOH group. In addition, EtOH exposure increased hepatic acetaldehyde accumulation (Figure 2I), which was also significantly decreased following PPE treatment. These data demonstrated that PPE alleviates EtOH‐induced hepatic injury, reduces serum TG levels, and lowers EtOH and acetaldehyde accumulation.

**FIGURE 2:**
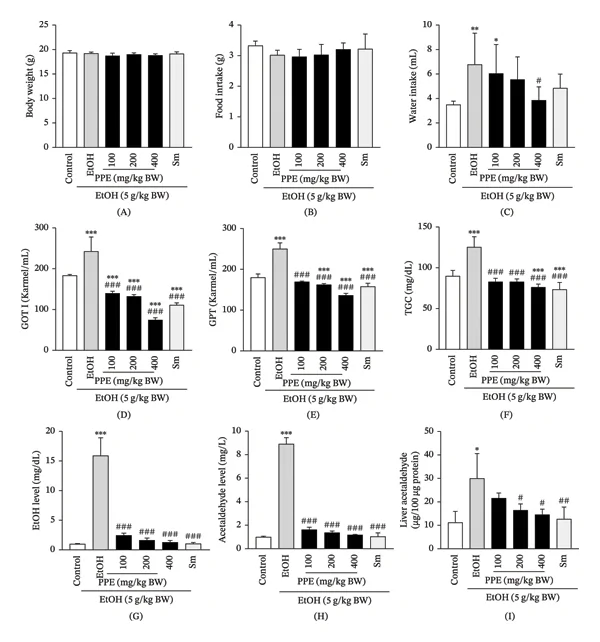
Effect of pear peel–enriched residue extract (PPE) on EtOH‐induced liver injury in mice. (A–C) Body weight and food and water intake. (D–F) Serum glutamate oxaloacetate transaminase (GOT), glutamate pyruvate transaminase (GPT), and triglyceride (TG) levels. (G, H) Ethanol and acetaldehyde levels. (I) Acetaldehyde in liver. Mice were treated with EtOH (5 g/kg BW) with or without PPE (100–400 mg/kg BW). Data are expressed as mean ± SD (*n* = 7). ^∗^ *p* < 0.05, ^∗∗^ *p* < 0.01, ^∗∗∗^ *p* < 0.001 versus control; ^#^ *p* < 0.05, ^##^ *p* < 0.01, ^###^ *p* < 0.001 versus EtOH group.

### 3.4. Histological Assessment of PPE‐Mediated Protection Against EtOH‐Induced Hepatic Steatosis and Tissue Injury

Oil Red O staining was performed to assess lipid accumulation and steatosis in liver tissues (Figure 3A). Minimal lipid deposition was observed in the control group. In contrast, liver sections from EtOH‐treated mice exhibited accumulation of red‐stained lipid droplets, indicating the development of macrovesicular steatosis due to alcohol exposure. PPE treatment markedly attenuated the EtOH‐induced hepatic lipid accumulation in a dose‐dependent manner. Notably, the group that received the highest PPE dose (400 mg/kg) showed a pronounced reduction in lipid droplet accumulation, with staining intensity comparable to that of the Sm‐treated group. These findings suggest that PPE exerts a dose‐dependent protective effect against alcohol‐induced hepatic steatosis by inhibiting neutral lipid accumulation in liver tissues.

**FIGURE 3:**
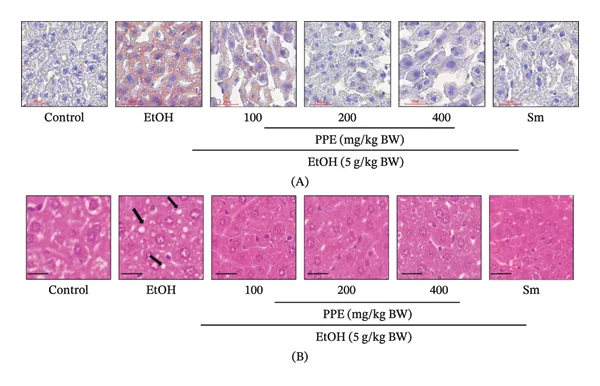
Histological analysis of liver tissues following EtOH exposure and PPE treatment. (A) Oil red O staining. (B) Hematoxylin and eosin (H&E) staining of liver tissue. Scale bar: 30 µm.

Histopathological analysis with H&E staining (Figure 3B) showed well‐preserved liver architecture in the control group, whereas the EtOH group exhibited significant hepatocellular damage, including ballooning degeneration, cytoplasmic vacuolation, and inflammatory cell infiltration (indicated by arrows). Administration of PPE ameliorated these alterations in a dose‐dependent manner, and the hepatic structure appeared largely normalized with decreased signs of degeneration and inflammation, supporting the hepatoprotective role of PPE. These observations support the efficacy of PPE in mitigating EtOH‐induced liver damage and preserving hepatic structural integrity.

### 3.5. PPE Modulates Protein Expression to Attenuate EtOH‐Induced Hepatic Lipotoxicity, Inflammation, and Oxidative Stress

EtOH administration significantly upregulated the hepatic expression of CYP2E1 by 2.8‐fold compared with the control group (*p* < 0.001), consistent with the enhanced oxidative metabolism of EtOH. Treatment with PPE reduced CYP2E1 expression in a dose‐dependent manner, with the 400 mg/kg group showing a 47% reduction compared with the EtOH group (*p* < 0.001), approaching the effect observed with silymarin. Lipogenic transcription factors were also elevated in EtOH‐exposed livers: SREBP1c and FAS increased by 2.4‐ and 2.7‐fold, respectively (*p* < 0.001). PPE treatment reversed these elevations, with a dose of 400 mg/kg reducing SREBP1c by 43% (*p* < 0.001) and FAS by 48% (*p* < 0.001) relative to EtOH treatment. EtOH exposure significantly increased total NF‐κB protein expression by 2.1‐fold relative to the control (*p* < 0.01), which was significantly attenuated by PPE. Similarly, Col1A1 expression, an early fibrogenic marker, increased 2.3‐fold in the EtOH group (*p* < 0.001), whereas treatment with PPE significantly suppressed this effect by 56% (*p* < 0.001). In contrast, Nrf2 expression was reduced by 41% in the EtOH group (*p* < 0.01 vs. control), reflecting an impaired antioxidant response. PPE restored Nrf2 levels in a dose‐dependent manner, with the 400 mg/kg group showing a 1.8‐fold increase compared with the EtOH group (*p* < 0.001). Likewise, GCLC, a downstream antioxidant enzyme, decreased by 38% in the EtOH group (*p* < 0.01) but was significantly upregulated by PPE, achieving a 1.7‐fold increase at 400 mg/kg (*p* < 0.001 vs. EtOH). Collectively, these data demonstrate that PPE exerts hepatoprotective effects against EtOH‐induced injury by repressing CYP2E1‐mediated oxidative stress, inhibiting lipogenic and early fibrogenic marker expression, and increasing the expression of antioxidant defenses‐related markers (Figure 4A,B).

FIGURE 4Protein expression analysis in liver tissues following EtOH exposure and PPE treatment. (A) Representative immunoblot images of hepatic protein expression levels. *β*‐Actin was used as a loading control. (B) Quantitative densitometric analysis of the respective protein bands normalized to *β*‐Actin. Data are expressed as mean ± SD (*n* = 3). ^∗^ *p* < 0.05, ^∗∗^ *p* < 0.01, ^∗∗∗^ *p* < 0.001 versus control; ^#^ *p* < 0.05, ^##^ *p* < 0.01, ^###^ *p* < 0.001 versus EtOH group.

**Figure:**
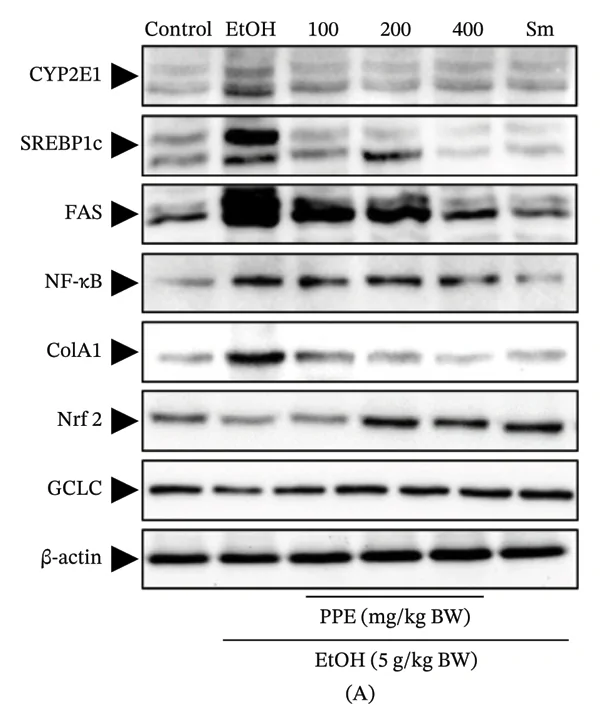

**Figure:**
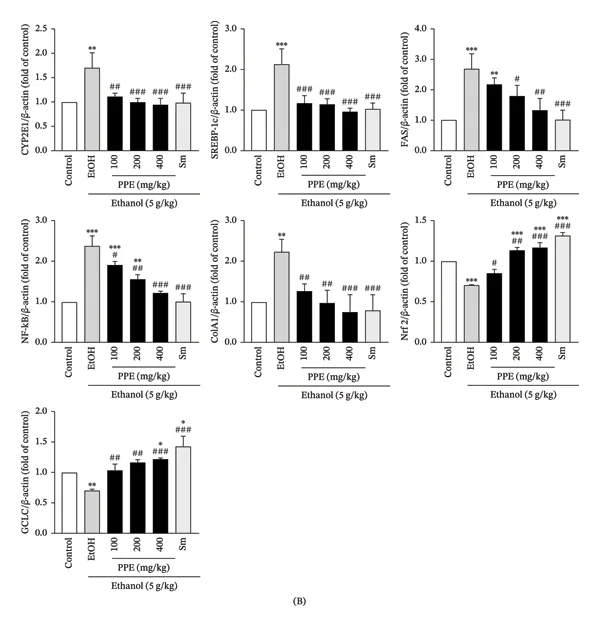

### 3.6. Putative Annotations of Compounds From the Extract

PPE compounds were analyzed using UHPLC–MS/MS dereplication to construct a molecular network‐based mass‐to‐charge ratio (m/z) of precursor ions, and their fragmentation patterns were obtained using tandem mass spectrometry (MS/MS) (Figure 5). This was achieved by calculating the similarity scores between spectra based on shared fragment ions. In the resulting network, each node signified a distinct molecular feature, while the connecting edges illustrated the extent of spectral similarity among predicted compounds. Compound annotation was executed via the GNPS–FBMN platform [22–24]. These predicted compounds were also annotated and supported using MS‐Dial [19] alongside publicly accessible databases. Analysis revealed a total of 120 compounds (Supporting Tables S1, S2, and Figure S2). Among these, alkaloids (21%) emerged as the most significant chemical class, followed by glycosides (11%), fatty acids (9%), terpenoids (9%), organic acids (7%), polyphenols (6%), and phenylpropanoids (5%) (Figure 6). Through the analysis of chromatographic peak areas, a range of metabolites was putatively annotated (Table 1; Supporting Table S1). These findings highlight their significant role in shaping the overall chemical profile of the extract. The fragmentation behavior of the isolated compounds was systematically evaluated using high‐resolution mass spectrometry (Supporting Table S3). Compound 6 generated a precursor ion [M+H]^+^ at *m*/*z* 266.1230 (C_10_H_20_NO_7_^+^), which subsequently yielded product ions at *m*/*z* 248 ((C_10_H_18_NO_6_^+^), 230 (C_10_H_17_NO_5_^+^), 146 (C_6_H_10_O_4_^+^) through the successive loss of water and a fructose unit; the 3‐carboxypropylamine moiety was further evidenced by the fragment at *m*/*z* 85 (C_4_H_7_O_2_^+^). Compound 7 displayed ions at *m*/*z* 147 (C_6_H_11_O_4_^+^), 133 (C_5_H_9_O_4_^+^), attributed to the simultaneous loss of water molecules and a methylene moiety [25]. In the case of Compound 26, the loss of a glucosyl moiety (C_6_H_11_O_6_) from the parent ion at *m*/*z* 328.1388 (C_15_H_18_O_7_NH_4_^+^) resulted in a prominent fragment at *m*/*z* 165.0756 (C_15_H_18_O_7_NH_4_^+^–C_6_H_11_O_6_) [26]. Compound 28 showed the protonated ion peak at *m*/*z* 166.0861 (C_12_H_12_NO_2_^+^) [27] and produced diagnostic fragments at *m*/*z* 147 (C_12_H_12_NO_2_^+^–H_2_O) and 120 (C_12_H_12_NO_2_^+^–COOH), representing the loss of water and COOH units, respectively.

**FIGURE 5:**
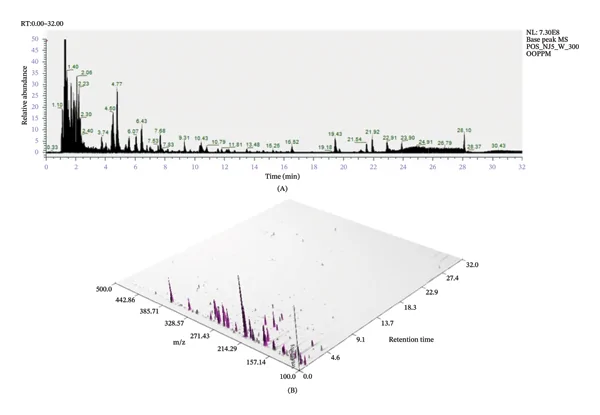
(A) 2D and (B) 3D chromatograms of the PPE detected in the positive ion mode.

**FIGURE 6:**
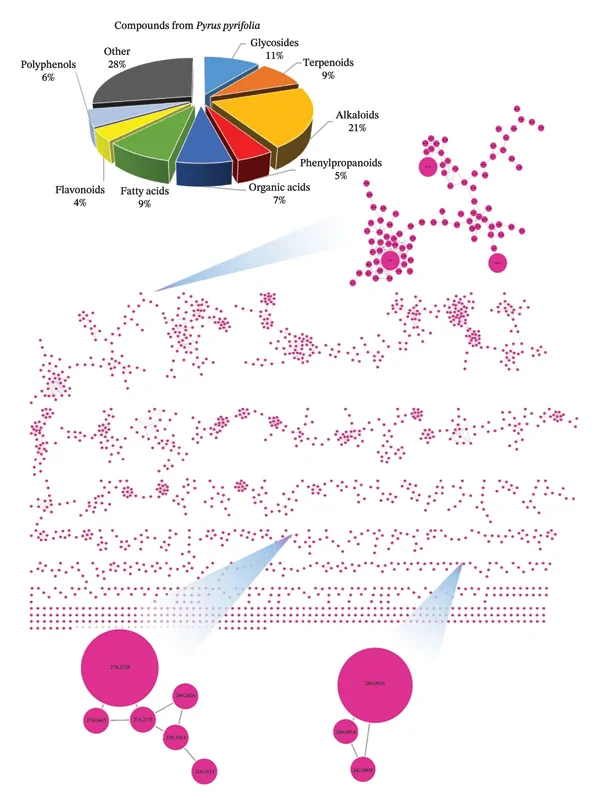
Visualization of the GNPS‐feature–based molecular network of the PPE.

**TABLE 1:** Chemicals and binding energy of major compounds.

Compounds from *Pyrus pyrifolia* peel extract								Binding energy (∆*G*, kcal/mol)			
								ADH	FAS	Liver fibrosis	ALDH
No.	Metabolites	RT (min)	MF	Adduct	M/Z	Error (mDA)	Class	1hdz	1zkt	3glu	4h80
3	2‐Hydroxy‐3,4‐dimethoxybenzoic acid	1.246	C_9_H_10_O_5_	[M+Na]^+^	221.0418	0.183	Organic acids	−8.24	−8.35	−7.32	−7.73
4	Gentiobiose	1.285	C_12_H_22_O_11_	[M+K]^+^	381.0787	0.641	Terpenoids	−9.99	−7.51	−9.15	−9.7
5	D‐Fructose	1.334	C_6_H_12_O_6_	[M+NH_4_]^+^	180.0864	0.000	Glycosides	−7.02	−6.88	−7.19	−6.30
6	D‐1‐[(3‐Carboxypropyl)amino]‐1‐deoxyfructose	1.3722	C_10_H_19_NO_7_	[M+H]^+^	266.1230	0.002	Alkaloids	−9.19	−8.72	−9.5	−7.96
7	D‐Sorbitol	1.42	C_6_H_14_O_6_	[M+H]^+^	183.0861	0.000	Glycosides	−6.65	−5.65	−6.54	−6.3
8	Mycosporine serinol	1.699	C_11_H_19_NO_6_	[M+H]^+^	262.1280	0.031	Phenylpropanoids	−9.66	−7.46	−7.4	−9.34
10	Tyrosine	1.894	C_9_H_11_NO_3_	[M+H]^+^	182.0808	0.351	Alkaloids	−7.89	−8.05	−7.47	−7.42
11	Noradrenaline	1.952	C_8_H_11_NO_3_	[M+H–H_2_O]^+^	152.0702	0.366	Alkaloids	−7.69	−7.15	−7.64	−6.76
12	Methyl 3,4,5‐trihydroxycyclohexene‐1‐carboxylate	1.962	C_8_H_12_O_5_	[M+ACN+H]^+^	230.1020	0.29	Phenylpropanoids	−7.79	−8.13	−8.19	−6.86
13	Norleucine	2.178	C_6_H_13_NO_2_	[M+H]^+^	132.10182	0.076	Alkaloids	−5.75	−6.63	−5.95	−5.04
14	Methoxyindoleacetic acid	2.25	C_11_H_11_NO_3_	[M+H]^+^	206.08096	0.9	Alkaloids	−8.26	−9.07	−8.42	−8.22
16	Hydroquinone	2.492	C^6^H^6^O^2^	[M+H]^+^	111.0439	0.114	Phenols	−5.56	−5.85	−5.43	−4.73
26	[(2*S*,3*R*,4*S*,5*S*,6*R*)‐3,4,5‐Trihydroxy‐6‐(hydroxymethyl)oxan‐2‐yl] (*E*)‐3‐phenylprop‐2‐enoate	3.74	C_15_H_18_O_7_	[M+NH_4_]^+^	328.13885	0.214	Glycosides	−12.24	−9.51	−10.24	−9.79
28	Phenylalanine	4.038	C_9_H_11_NO_2_	[M+H]^+^	166.0861	0.137	Alkaloids	−4.60	−8.04	−7.11	−3.24
30	Pyrogallol	4.431	C_6_H_6_O_3_	[M+H]^+^	127.0389	0.114	Phenols	−6.2	−6.23	−6.03	−5.41
31	N‐Acetylleucine	4.453	C_8_H_15_NO_3_	[M+H]^+^	196.0966	0.246	Fatty Acids	−4.28	−7.73	−6.82	−4.39
32	Pantothenic acid	4.495	C_9_H_17_NO_5_	[M+H]^+^	220.1176	0.32	Alkaloids	−8.78	−9.81	−8.57	−8.05
35	Methyl asterate	5.586	C_18_H_18_O_8_	[M+NH_4_]^+^	380.1337	0.336	Phenols	−5.78	−9.06	9.76	−2.76
36	4,7,8‐Trimethoxyfuro[2,3‐b]quinoline	5.999	C_14_H_13_NO_4_	[M+H]^+^	260.0915	0.244	Alkaloids	−8.41	−7.64	−8.14	−8.8
37	5‐Phenyllevulinic acid	6.062	C_11_H_12_O_3_	[M+NH_4_]^+^	210.1122	0.244	Organic acids	−5.71	−9.14	−7.56	−6.21
77	Hydroxyphenyllactate	9.31	C_11_H_8_O_4_	[M+Na]^+^	205.0493	0.2493	Ketones	−6.75	−8.25	−7.61	−6.77
85	Perillyl alcohol	10.432	C_10_H_16_O	[M+H]^+^	153.1272	0.229	Terpenoids	−6.52	−7.64	−6.61	−6.47
117	(9Z)‐5,8,11‐Trihydroxyoctadec‐9‐enoic acid	16.525	C_18_H_34_O_5_	[M+H]+	353.2294	1.791	Fatty acids	−4.00	−6.21	−8.74	−5.29
118	Lauryldiethanolamine	19.443	C_16_H_35_NO_2_	[M+H]^+^	274.2735	0.519	Alkaloids	−6.48	−5.32	−6.83	−6.74
119	Gelomulide N	21.54	C_24_H_32_O_7_	[M‐H_2_O+H]^+^	415.2109	0.61	Naphthofurans	−10.96	−10.05	−11.72	−6.93

*Note:* ADH, alcohol dehydrogenase. ALDH, aldehyde dehydrogenase.

Abbreviation: FAS, fatty acid synthase.

A common dehydration pathway was observed for several other compounds. Specifically, Compounds 8, 12, 30, 32, 85, and 118 yielded fragment ions at *m*/*z* 244 (C_11_H_18_NO_5_^+^) [28], 212 (C_10_H_15_NO_4_^+^) [29], 109 (C_6_H_5_O_2_^+^) [30], 202 (C_9_H_16_NO_4_^+^) [31], 135 (C_10_H_15_^+^) [32], and 256 (C_16_H_34_NO^+^) [33], respectively, each corresponding to the loss of a water molecule from their protonated molecular ions. Finally, Compound 10 underwent specific cleavage at the carboxylic and amino sites, producing characteristic fragments at m/z 136 (C_8_H_10_NO^+^) and 119 (C_8_H_7_NO^+^), consistent with previous reports [34]. Compounds 31 and 36 demonstrated a protonated ion peak at *m*/*z* 196.0966 (C_8_H_15_NO_3_Na^+^) [29] and *m*/*z* 260.0915 (C_14_H_14_NO_4_^+^) [35], respectively. At the same time, they demonstrated a fragmentation pattern due to the reduction of CH_3_ and a water molecule at *m*/*z* 183.0861 (C_12_H_12_NO_2_–CH_3_^+^) and at *m*/*z* 180 (C_12_H_12_NO_2_–H_2_O^+^) (for 31) and at *m*/*z* 245.0587 (C_13_H_10_NO_4_–CH_3_^+^) and at *m*/*z* 227.0911 (C_13_H_10_NO_3_^+^) for 36. Compound 35 showed a protonated ion peak at *m*/*z* 380.1336 (C_18_H_18_O_8_NH_4_^+^) and fragmented into *m*/*z* 262.1071 (C_18_H_18_O_8_NH_4_–C_4_H_6_O_4_^+^) consistent with an elimination of two methyl ester groups [36]. Compound 77 showed a sodiated ion peak at *m*/*z* 205.0493 (C_9_H_10_O_4_Na^+^) and it produced a fragment peak at *m*/*z* 145.0494 (C_9_H_10_ONa^+^) due to the eliminating of COOH and water molecules [37]. Compound 117 presented a parent ion peak at *m*/*z* 353.2294 (C_18_H_34_O_5_Na^+^). At the same time, it may produce a fragment at *m*/*z* 296.2301 (C_18_H_32_O_3_^+^) involving the elimination of two water molecules [38].

### 3.7. Molecular Docking Analysis

To assess the molecular interactions of selected compounds and target proteins, we evaluated their binding affinity in the binding sites of each protein. *In silico* results revealed that selected metabolites exhibited distinct affinities toward four key enzymes, ADH, FAS, 3GLU, and ALDH, which play important roles in alcohol metabolism, lipid synthesis, and early fibrogenesis. The selected metabolites docked to the ADH protein at the same location in the active site of the protein (Figure 7). Among them, Compounds 4, 26, and 119 displayed high binding affinities through low values of docked functional scores of −9.99, −12.24, and −10.96 kcal/mol, respectively. Other ligands also demonstrated good binding (docked scores ranged from −6.20 to −9.99 kcal/mol). Notably, Compounds 3–6, 8, 11, 13, 16, 26, 32, 36, 77, 85, and 119 interacted with key residues (VAL294) at the active site of the protein [24]. Furthermore, Compounds 4 and 26 interacted with GLY204 and VAL268 through hydrogen bonds. Similarly, Compound 36 also showed interactions with CYS46 and ARG369 residues (Supporting Figure S3), which are occupied at the binding region of the protein. This suggests these compounds may influence ADH activity. For the FAS thioesterase domain (Figure 8) [39], Compound 119 showed the lowest docked score of −10.05 kcal/mol and showed strong interaction with the key SER2308 residue [24] through hydrogen bonds. Compounds 3, 6, 10, 12, 14, 26, 28, 32, 35, 37, and 77 also exhibited good binding affinity, showing docking scores in the range of −8.05 to −9.81 kcal/mol. Compounds 4–7, 10, 13, 16, 26, 28, 30–32, and 77 interacted with LEU2222 via hydrogen bonds. Compounds 3, 5, 10, 16, 30, and 35 showed hydrogen bonding interactions with ILE2250. In addition, Compounds 4, 6, 10, and 77 interacted with GLN2374 by forming hydrogen bonds (Supporting Figure S4). Compounds 26, 28, 32, and 117 formed hydrogen bonds with the amino acids ALA2419, GLU2251, and SER2422. When they were docked to 3GLU protein (Figure 9), Compounds 26, 35, and 119 exhibited low binding energy Δ*G* = −10.24, −9.76, and −11.72 kcal/mol, respectively. These compounds formed hydrogen bonds with VAL292 and ALA146 residues (Supporting Figure S5). The other compounds showed binding affinities ranging from −5.43 to −9.5 kcal/mol. When they were docked to the ALDH protein (Figure 10), Compounds 4, 8, and 26 exhibited low docking scores of −9.70, −9.34, and −9.79 kcal/mol, respectively. Compound 4 interacted with TYR60, GLU61, and ASN118 by forming hydrogen bonds (Supporting Figure S6). Compound 8 showed hydrogen bonding interactions with the amino acids ASN114 and ASN118. Compound 26 formed hydrogen bonds with GLU61, TYR65, and TYR115 amino acids [40]. Other compounds exhibited binding energies ranging from −2.76 to −8.80 kcal/mol (Table 1). The selected compounds interacted with target proteins with favorable binding affinities, supporting their potential relevance to liver injury‐related targets. These docking results are hypothesis generating and indicate computational binding compatibility only and should be interpreted as a basis for future studies.

**FIGURE 7:**
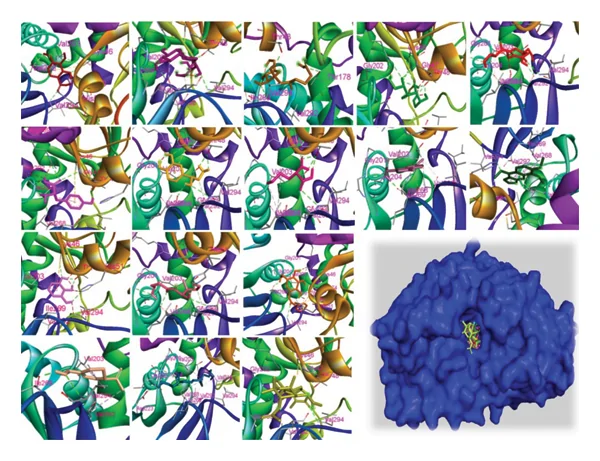
3D visualization of ligands [ruby (3), deep purple (4), sand (6), lime green (7), red (8), magenta (10), orange (11), hot pink (13), raspberry (14), forest (26), violet (30), salmon (32), tv‐orange (36), wheat [85], sky blue (118), and limon (119)] that interacted with the 1HDZ protein.

**FIGURE 8:**
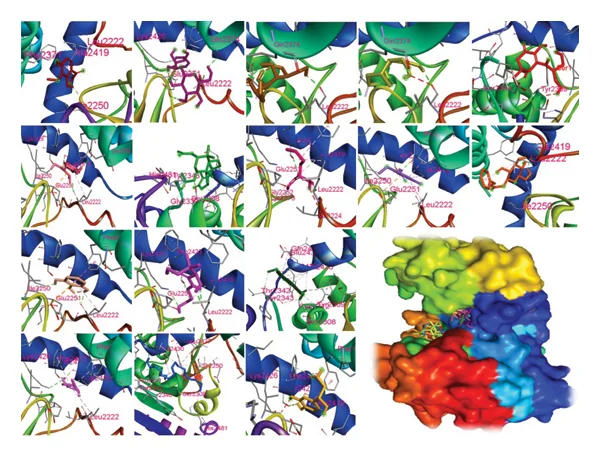
3D visualization of ligands [ruby (3), deep purple (4), sand (6), lime green (7), red (8), magenta (10), orange (11), hot pink (13), raspberry (14), forest (26), violet (30), salmon (32), tv‐orange (36), wheat [85], sky blue (118), and limon (119)] that interacted with the 1XKT protein.

**FIGURE 9:**
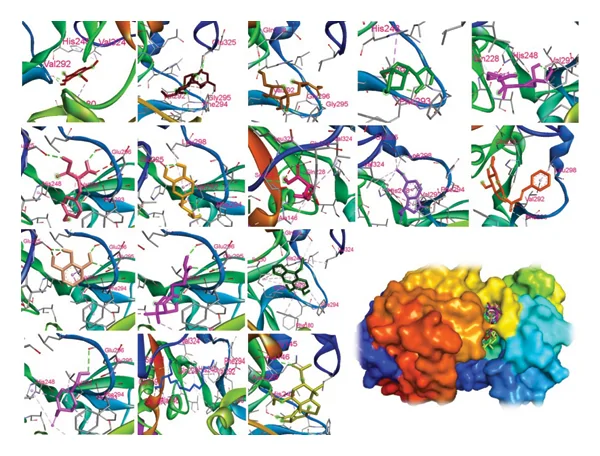
3D visualization of ligands [ruby (3), deep purple (4), sand (6), green (7), red (8), magenta (10), lime green (11), hot pink (13), violet (14), forest (26), wheat (30), magenta (32), lime (36), warm pink (85), sky blue (118), and limon (119)] that interacted with the 3GLU protein.

**FIGURE 10:**
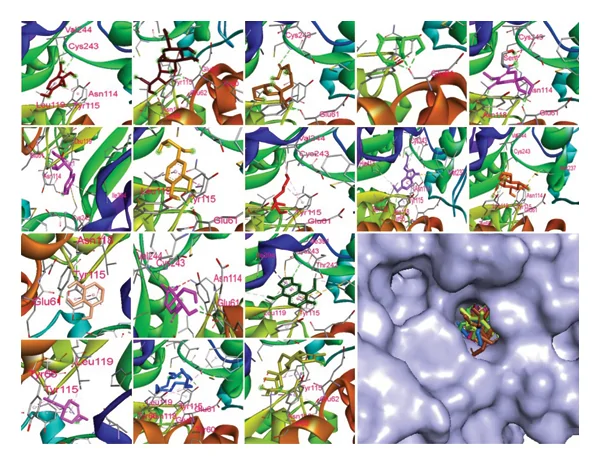
3D visualization of ligands [ruby (3), deep purple (4), sand (6), green (7), deep purple (8), magenta (10), orange (11), red (13), violet (14), brown (26), wheat (30), hot pink (32), forest (36), raspberry (85), marine (118), and limon (119)] that interacted with the 4H80 protein.

### 3.8. MD Simulations

To investigate the structural dynamics and stability of selected metabolites and proteins relevant to liver injury under various conditions, we performed MD simulations of compounds showing strong binding to the aforementioned target proteins. Key parameters, including the RMSD, SASA, Rg, number of hydrogen bonds (H‐bonds), and RMSF, were analyzed to assess the conformational changes, compactness, and stability of the established ligand–protein complexes (Figure 11).

**FIGURE 11:**
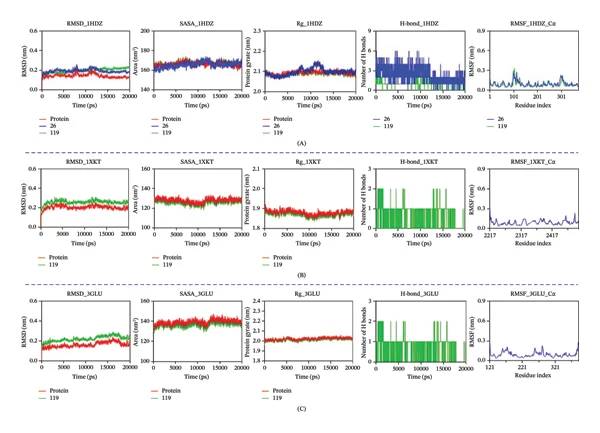
Molecular dynamics simulation parameters (RMSD, SASA, H‐bond, Rg) of strong active compounds binding to proteins: 1HDZ (A), 1XKT (B), and 3GLU (C) targeted liver injuries.

### 3.9. RMSD Analysis

The RMSD profiles of complexes with ADH, FAS, and liver fibrosis proteins demonstrated initial equilibration at the beginning of the simulation, followed by a relatively stable trajectory. These ligand–protein complexes displayed RMSD values less than 0.30 nm. The RMSD values for the protein and complexes with Compounds 26 and 119 indicated distinct dynamic behavior (Figure 11). In the ADH system, Compounds 26 and 119 exhibited RMSD values of approximately 0.16–0.25 and 0.18–0.24 nm, respectively, slightly higher compared with those of the protein (RMSD: 0.16–0.22 nm). This suggests that the presence of a ligand may affect the stable conformation of the complexes compared with that of the original proteins. Similarly, for the FAS system and liver fibrosis protein, the ligand–protein complex also exhibited a higher RMSD value (0.17–0.28 nm) compared with that of the original protein (RMSD: 0.16–0.23 nm). The Compound 119 complex also exhibited RMSD values of approximately 0.19–0.27 nm compared with those of 3GLU protein (RMSD: 0.14–0.20 nm). This observation indicates relative stability of the complexes during simulation.

### 3.10. SASA Analysis

SASA values reflect the extent of the solvent exposure of proteins. The Compound 119‐ADH complex showed a lower SASA value (158–169 nm^2^) than that of the protein, suggesting that this compound may shield solvent exposure after binding to the protein. The Compound 26 complex and ADH protein displayed similar SASA values, indicating that the binding of this compound did not affect the solvent exposure of this protein. Both FAS and liver fibrosis proteins showed a small decrease in SASA upon binding of Compound 119, suggesting that the buried surface area increased in the complexes compared with that in the apo form. Conversely, in all three protein systems, SASA remained relatively consistent across all conditions, suggesting a stabilized conformation upon binding.

### 3.11. Rg

Rg provides insights into protein compactness. Both FAS and liver fibrosis proteins demonstrated lower Rg values upon binding to Compound 119. This observation is consistent with their SASA values, indicating that Compound 119–protein complexes were more compact and potentially more stably structured in the ligand‐bound state during the simulation. The 26‐ADH complex displayed some fluctuations during the simulation time of 7–13 ns. This observation is consistent with the slight fluctuations in the SASA plot during the respective frame times.

### 3.12. Hydrogen Bond Analysis

The number of hydrogen bonds formed between the protein and the solvent/ligand is indicative of structural stability. The ADH system showed varying numbers of hydrogen bonds between the proteins and ligands. Compound 26 showed up to 6 hydrogen bonds, while Compound 119 showed up to 2 hydrogen bonds. Similarly, the H‐bond profiles of the FAS and liver fibrogenic‐related proteins showed similar trends across all simulations, suggesting that the presence of Compound 119 did not significantly affect the number of hydrogen bonds formed.

### 3.13. RMSF analysis

The RMSF plots revealed the average deviation of C‐alpha residues of ligand–protein complexes during simulation time. Compounds 26 and 119 provided significant stabilization to their respective protein complexes with a fluctuation of C‐α atoms below 0.33 nm. Particularly, both compounds revealed higher flexibility near residues 97–120 and 293–311 with RMSF values in the ranges of 0.04–0.33 nm. Furthermore, the data for 1XKT and 3GLU complexes demonstrate that Compound 26 maintains a consistently high level of rigidity (mostly under 0.2 nm) across different protein targets (Figure 11). Overall, the low and stable RMSF values indicate that both ligands form stable complexes without inducing significant structural distortions and have robust performance across all tested environments.

### 3.14. ADME Analysis

The admetSAR 3.0 web platform [41] was utilized to predict the in silico ADMET properties of the primary components found in *P. pyrifolia* extract. Moreover, a comprehensive assessment was conducted, incorporating essential parameters such as HIA, Caco‐2 permeability, blood–brain barrier (BBB) penetration, PPB, human liver microsome (HLM) stability, DILI, bioavailability profiles, and Ames mutagenicity. Table S4 (absorption and distribution) and Table S5 (metabolism and toxicity) provide an overview of the findings.

Concerning absorption, a substantial number of the Compounds 11 of the 20 selected compounds (55%), demonstrated elevated HIA values, exceeding 80%. These predictions indicate relatively favorable predicted intestinal absorption for several compounds; however, experimental pharmacokinetic validation is required. The compounds demonstrating the most substantial HIA values, exceeding 95%, included 4,7,8‐trimethoxyfuro[2,3‐b]quinoline (36), perillyl alcohol (85), methyl asterrate (35), and lauryldiethanolamine (118). Furthermore, methoxyindoleacetic acid (14), 5‐phenyllevulinic acid (37), and 2‐hydroxy‐3,4‐dimethoxybenzoic acid (3) all exhibited high HIA values of 94.3%, 90.7%, and 90.5%, respectively. Conversely, polar sugar‐based compounds like gentiobiose (4), D‐1‐[(3‐carboxypropyl)amino]‐1‐deoxyfructose (6), and D‐sorbitol (7) showed very low HIA values (< 20%), which is consistent with their poor intestinal permeability and high hydrophilicity. These computational findings are predictive in nature and do not substitute for experimental evidence; direct toxicological validation is therefore necessary to confirm safety or genotoxic potential.

In terms of metabolic stability, the majority of compounds showed acceptable stability against hepatic microsomal metabolism with low HLM values (< 30%). In particular, the lowest HLM percentages were found in methoxyindoleacetic acid (2.7%), tyrosine (0.8%), 2‐hydroxy‐3,4‐dimethoxybenzoic acid (2.9%), norleucine (1.7%), and (9Z)‐5,8,11‐trihydroxyoctadec‐9‐enoic acid (2.6%), indicating minimal first‐pass hepatic metabolism. On the other hand, HLM values for lauryldiethanolamine (67.2%), methyl asterrate (49.4%), and gelomulide N (36.6%) were significantly higher, suggesting rapid microsomal metabolism.

In terms of toxicity, it was estimated that 15 out of 20 compounds (75%) would be safe in terms of DILI. Five substances were identified as possible DILI risks: pyrogallol (30), cinnamoyl glucoside (26), 4,7,8‐trimethoxyfuro[2,3‐b]quinoline (36), lauryldiethanolamine (118), and gelomulide N (119). Although pyrogallol (30), 4,7,8‐trimethoxyfuro[2,3‐b]quinoline (36), lauryldiethanolamine (118), and gelomulide N (119) yielded positive Ames results, thereby suggesting potential genotoxicity, Ames mutagenicity testing indicated that 16 compounds (80%) were nonmutagenic. Therefore, the putatively annotated compounds 2‐hydroxy‐3,4‐dimethoxybenzoic acid (3), methoxyindoleacetic acid (14), 5‐phenyllevulinic acid (37), hydroxyphenyllactate (77), and (9Z)‐5,8,11‐trihydroxyoctadec‐9‐enoic acid (117) exhibited the most favorable combined ADMET profiles.

## 4. Discussion

This study demonstrates that the PPE exerts protective effects against EtOH‐induced hepatic injury through modulation of oxidative stress, lipid dysregulation, and inflammatory signaling. In the present study, two pear‐derived extracts were evaluated: PE, obtained from the juice fraction, and PPE, prepared from the pear peel‐rich residue remaining after juice extraction. By integrating extract characterization, cellular assays, animal experiments, and molecular analyses, the present work provides a comprehensive evaluation of the hepatoprotective potential of PPE in models of alcohol‐induced liver injury.

Initial antioxidant profiling revealed that PPE contained substantially higher total phenolic content than PE, accompanied by stronger DPPH radical‐scavenging activity (Supporting Figure S1). This phenolic enrichment suggests that the peel fraction serves as a concentrated source of antioxidant‐associated phytochemicals in *P. pyrifolia*. Phenolic compounds are well recognized for their ability to neutralize ROS and regulate redox‐sensitive signaling pathways, which are key contributors to EtOH‐induced hepatic damage [42]. Therefore, the higher antioxidant capacity observed for PPE provides a biochemical basis for its enhanced cytoprotective effects in subsequent cellular assays.

In HepG2 cells, exposure to 3% EtOH markedly induced cytotoxicity and oxidative stress, as indicated by reduced cell viability and increased intracellular ROS levels. Treatment with PE and PPE significantly restored cell viability and attenuated ROS accumulation in a dose‐dependent manner. Notably, PPE exerted stronger protective effects than PE and showed activity comparable to that of the reference hepatoprotectant, silymarin. PPE also normalized EtOH and acetaldehyde levels, key metabolites generated during EtOH metabolism. These findings suggest that PPE reduces oxidative stress and is associated with lower intracellular EtOH and acetaldehyde accumulations; however, because ADH and ALDH enzymatic activities were not directly measured, this reduction may instead reflect decreased oxidative burden or altered cellular EtOH metabolism or its metabolite level. The superior activity of PPE compared with PE is consistent with previous reports indicating that PPE contains higher levels of antioxidant compounds and exhibits stronger radical‐scavenging activities [43], reinforcing the importance of peel‐associated phytochemicals.

Consistent with the in vitro findings, PPE supplementation in EtOH‐treated mice significantly reduced serum liver injury markers (GOT and GPT), decreased TG accumulation, and improved histopathological features of hepatic injury. Oil Red O staining demonstrated reduced hepatic lipid deposition, while H&E staining showed improvements in EtOH‐associated morphological alterations such as hepatocellular ballooning, cytoplasmic vacuolation, and inflammatory infiltration. The reductions in serum GOT and GPT levels, together with the improved histological appearance of liver tissues, support attenuation of EtOH‐induced hepatic injury following PPE treatment. At the molecular level, PPE downregulated CYP2E1 expression, a cytochrome P450 isoenzyme that enhances ROS production and acetaldehyde formation during EtOH metabolism [44, 45]. In addition, CYP2E1‐derived ROS has been reported to disrupt lipid homeostasis and stimulate lipogenic signaling pathways [46] by activating transcription factors such as SREBP‐1c and its downstream enzyme FAS, thereby promoting TG synthesis and lipid accumulation in hepatocytes [47]. Consistent with this mechanism, PPE treatment significantly reduced the expression of SREBP‐1c and FAS, suggesting suppression of EtOH‐induced hepatic lipogenesis. Moreover, oxidative stress and lipid accumulation are known to activate inflammatory signaling pathways, particularly NF‐κB [48], which regulate the expression of proinflammatory mediators and contribute to the progression of alcoholic liver injury and fibrosis [49]. The observed reduction of NF‐κB expression in PPE‐treated mice therefore indicates attenuation of inflammatory responses associated with EtOH exposure. Furthermore, PPE decreased the expression of Col1A1, an early profibrogenic ECM marker [50, 51], suggesting attenuation of early fibrogenic signaling associated with EtOH‐induced liver injury. Importantly, PPE also upregulated the expression of NRF2 and its downstream effector, GCLC, key regulators of cellular antioxidant defense [52], indicating restoration of endogenous antioxidant signaling and maintenance of redox homeostasis. Nonetheless, the study findings indicate changes in overall protein expression rather than the direct confirmation of NF‐κB pathway activation or NRF2 nuclear translocation, where future studies would be needed to confirm the activation status.

To investigate active compounds potentially responsible for these effects, UHPLC–MS/MS‐based metabolite profiling putatively annotated over 120 phytochemicals in PPE, including alkaloids, glycosides, terpenoids, and phenylpropanoids. Docking simulations showed that several of these compounds (notably peaks 4, 26, and 119) exhibited strong binding affinities to liver injury‐related targets, such as ADH, ALDH, FAS, and fibrosis‐related proteins. For example, Compound 26 formed stable hydrogen bonds with GLY204 and VAL268 of ADH and SER2308 within the FAS thioesterase domain. These in silico results suggest potential interactions between PPE constituents and key enzymes. Furthermore, MD simulations supported the computational stability of these ligand–protein complexes, highlighting PPE as a potential source of bioactive compounds. Computational ADMET analysis of the main components of P. pyrifolia provided preliminary hypothesis‐generating insights into the pharmacokinetic and safety profiles of selected metabolites. Importantly, several metabolites also showed predicted DILI and/or Ames mutagenicity signals. Therefore, the computational ADMET profiles should not be interpreted as confirmation of pharmacokinetic suitability or safety and require experimental pharmacokinetic and toxicological validation. The hepatoprotective properties of the extract are likely associated with its ability to regulate oxidative stress, lipid metabolism, inflammation, and fibrogenic responses. ADMET analysis has identified several metabolites of *P. pyrifolia*, including methoxyindoleacetic acid, 2‐hydroxy‐3,4‐dimethoxybenzoic acid, 5‐phenyllevulinic acid, hydroxyphenyllactate, and (9Z)‐5,8,11‐trihydroxyoctadec‐9‐enoic acid, as potential subjects for further research. The diverse activity profile of the extract, encompassing antioxidant, anti‐inflammatory, and lipid‐regulatory effects, may reflect the combined contribution of its multiple constituents acting through different or potentially complementary pathways.

In summary, PPE demonstrated protective effects against EtOH‐induced liver injury, associated with attenuation of oxidative stress, suppressing hepatic lipogenesis, and reducing inflammatory and early fibrogenic responses. These effects were associated with decreased CYP2E1, SREBP‐1c, FAS, NF‐κB, and Col1A1 expression, together with restoration of NRF2/GCLC‐mediated antioxidant signaling. Metabolite profiling revealed that PPE contains diverse phytochemicals, and complementary docking, MD, and ADMET analyses suggested that several metabolites may potentially contribute to its hepatoprotective activity through interactions related to EtOH‐associated cellular stress, lipid regulation, and redox homeostasis; however, these computational predictions require direct experimental validation and should not be interpreted as a confirmed mechanism of action. Taken together, these findings suggest that PPE may serve as a candidate phenolic‐enriched resource warranting further investigation for the potential mitigation of alcohol‐induced liver injury. Several limitations of the present study should be acknowledged. The use of the HepG2 cell line, while widely used for hepatotoxicity studies, does not fully recapitulate the metabolic and functional complexity of primary human hepatocytes. Besides, the histological observations in this study were qualitative, and future studies incorporating quantitative scoring would strengthen these findings. This study relied on serum enzyme markers (GOT/GPT) but did not include direct enzyme‐activity assays, which could provide stronger mechanistic support. Furthermore, the computational findings from in silico analysis are predictive in nature and require direct experimental validation, including enzymatic assays, pathway activation studies, and pharmacokinetic analyses. Finally, while this study focused primarily on the hepatoprotective mechanism, the systemic effects of PPE on other organs such as the kidney, intestine, lungs, and heart remain to be evaluated; future studies incorporating histopathological and biochemical assessment of these tissues would provide a more comprehensive characterization of PPE safety. Additionally, gut microbiota‐mediated biotransformation of ingested polyphenols in vivo may further modulate the bioavailability and hepatoprotective efficacy of PPE constituents, representing an important avenue for future investigations.

## Author Contributions

Laxmi Sen Thakuri and Ki‐Sun Lee conceptualized the study. Laxmi Sen Thakuri, Ducdat Le, Narayan Sah Sonar, and Hye Bin Park performed formal analyses. Laxmi Sen Thakuri, Ducdat Le, Narayan Sah Sonar, Hye Bin Park, Laxman Subedi, and Jeong‐Ho Shon carried out the investigations. Ki‐Sun Lee and Jeong‐Ho Shon provided resources. Laxmi Sen Thakuri and Ducdat Le prepared the visualizations. Jin Woo Park, Mina Lee, and Dong Young Rhyu supervised the work. Laxmi Sen Thakuri and Ducdat Le drafted the original manuscript. Laxmi Sen Thakuri, Ducdat Le, and Dong Young Rhyu reviewed and edited the manuscript.

## Funding

This work was supported by the Basic Science Research Program, National Research Foundation of Korea with project No. RS‐2022‐NR070862, Republic of Korea, as well as supported by Korea Basic Science Institute (National Research Facilities and Equipment Center) grant funded by the Ministry of Education (RS‐2026‐25533557), Republic of Korea. The authors also acknowledge the Core Research for Support AI Bio Autonomous Research Core Facility Center for technical support regarding high‐sensitivity nanoflow rate liquid chromatograph (NFEC‐2025‐03‐304724).

## Disclosure

The final version of the manuscript was read and approved by all authors.

## Conflicts of Interest

The authors declare no conflicts of interest.

## Supporting Information

Additional supporting information can be found online in the Supporting Information section.

## Supporting information


**Supporting Information 1** Table S1. Identification of components from *P. pyrifolia* extract. Table S2. Experimental and reference spectra of compound annotations. Table S3. Tentative identification of major chemical constituents of *P. pyrifolia* extract in the positive polarity. Table S4. Predicted absorption and distribution parameters of identified metabolites from *P. pyrifolia.* Table S5. Predicted metabolism and toxicity parameters of identified metabolites from *P. pyrifolia.*

**Supporting Information 2** Figure S1. Antioxidant characterization of pear‐derived extracts. (A) Total phenolic content (TPC). (B) DPPH‐radical scavenging activity. Figure S2. Relative content of detected compounds from PPE based on calculation of peak areas. Figure S3. Interactions of selected compounds with binding sites of the 1HDZ protein. Figure S4. Interactions of selected compounds with binding sites of the 1XKT protein. Figure S5. Interactions of selected compounds with binding sites of the 3GLU protein. Figure S6. Interactions of selected compounds with binding sites of the 4H80 protein.

## Data Availability

Data will be available on request from the corresponding author.

## References

[1] Mackowiak B. , Fu Y. , Maccioni L. , and Gao B. , Alcohol-Associated Liver Disease, Journal of Clinical Investigation. (2024) 134, no. 3, 10.1172/JCI176345. PMC10836812 38299591

[2] Hernandez-Tejero M. , Clemente-Sanchez A. , and Bataller R. , Spectrum, Screening, and Diagnosis of Alcohol-Related Liver Disease, Journal of Clinical and Experimental Hepatology. (2023) 13, no. 1, 75–87, 10.1016/j.jceh.2022.10.002. PMC9840079 36647416

[3] Yunusoğlu O. , Resveratrol Impairs Acquisition, Reinstatement and Precipitates Extinction of Alcohol-Induced Place Preference in Mice, Neurological Research. (2021) 43, no. 12, 985–994, 10.1080/01616412.2021.1948749. 34210247

[4] Arab J. P. , Roblero J. P. , Altamirano J. et al., Alcohol-Related Liver Disease: Clinical Practice Guidelines by the Latin American Association for the Study of the Liver (ALEH), Annals of Hepatology. (2019) 18, no. 3, 518–535, 10.1016/j.aohep.2019.04.005. 31053546

[5] Zakhari S. , Overview: How is Alcohol Metabolized by the Body?, Alcohol Research & Health. (2006) 29, no. 4, 245–254. PMC6527027 17718403

[6] Cederbaum A. I. , Alcohol Metabolism, Clinics in Liver Disease. (2012) 16, no. 4, 667–685, 10.1016/j.cld.2012.08.002. PMC3484320 23101976

[7] Oidovsambuu S. , Yun J. H. , Kang K. et al., A Fruit Extract of *Paeonia anomala* Attenuates Chronic Alcohol-Induced Liver Damage in Rats, Natural Product Sciences. (2016) 22, no. 4, 231–237, 10.20307/nps.2016.22.4.231.

[8] Yan C. , Hu W. , Tu J. , Li J. , Liang Q. , and Han S. , Pathogenic Mechanisms and Regulatory Factors Involved in Alcoholic Liver Disease, Journal of Translational Medicine. (2023) 21, no. 1, 10.1186/s12967-023-04166-8. PMC10158301 37143126

[9] Braud L. , Battault S. , Meyer G. et al., Antioxidant Properties of Tea Blunt ROS-Dependent Lipogenesis: Beneficial Effect on Hepatic Steatosis in a High Fat-High Sucrose Diet NAFLD Obese Rat Model, The Journal of Nutritional Biochemistry. (2017) 40, 95–104, 10.1016/j.jnutbio.2016.10.012. 27866076

[10] Seo E. , Nam H. , and Jun H. S. , Reactive Oxygen Species Induce HNF-4α Expression via the ASK1-CREB Pathway, Promoting ChREBP Expression and Lipogenesis in Hepatocytes, Life Sciences. (2022) 310, 10.1016/j.lfs.2022.121042. 36208661

[11] Cho H. J. , Jang J. Y. , and Kim T. M. , The Role of Hepatic Stellate Cells in Fibrotic Liver Diseases, Journal of Animal Reproduction and Biotechnology. (2020) 35, no. 2, 113–118, 10.12750/jarb.35.2.113.

[12] Gao B. and Bataller R. , Alcoholic Liver Disease: Pathogenesis and New Therapeutic Targets, Gastroenterology. (2011) 141, no. 5, 1572–1585, 10.1053/j.gastro.2011.09.002. PMC3214974 21920463

[13] Qadri S. S. , Javaid D. , Reyaz A. , Ganie S. Y. , and Reshi M. S. , Liver Disorders and Phytotherapy, Toxicology Reports. (2025) 14, 10.1016/j.toxrep.2025.102047. PMC12142353 40487380

[14] Zhang C. Y. , Liu S. , and Yang M. , Antioxidant and Anti-Inflammatory Agents in Chronic Liver Diseases: Molecular Mechanisms and Therapy, World Journal of Hepatology. (2023) 15, no. 2, 180–200, 10.4254/wjh.v15.i2.180. PMC10011909 36926234

[15] Yunusoğlu O. , Ayaz İ. , and Dovankaya E. H. , Pharmacological, Medicinal and Biological Properties of Flavonoids: A Comprehensive Review, Journal of Research in Pharmacy. (2025) 29, no. 2, 561–584, 10.12991/jrespharm.1661054.

[16] Hong S. Y. , Lansky E. , Kang S. S. , and Yang M. , A Review of Pears (*Pyrus* spp.), Ancient Functional Food for Modern Times, BMC Complementary Medicine and Therapies. (2021) 21, no. 1, 10.1186/s12906-021-03392-1. PMC8409479 34470625

[17] Lee H. S. , Isse T. , Kawamoto T. et al., Effects and Action Mechanisms of Korean Pear (*Pyrus pyrifolia* cv. Shingo) on Alcohol Detoxification, Phytotherapy Research. (2012) 26, no. 11, 1753–1758, 10.1002/ptr.4630. 22451246

[18] Savic A. , Aradski A. A. , Zivkovic J. et al., Phenolic Composition and Antioxidant Potential of Pear Peel and Flesh Extracts, Polish Journal of Food and Nutrition Sciences. (2021) 71, no. 2, 225–236, 10.31883/pjfns/138320.

[19] Le D. D. , Jang Y. S. , Truong V. et al., Anti-Inflammatory Effects and Metabolomic Analysis of *Ilex rotunda* , International Journal of Molecular Sciences. (2024) 25, no. 22, 10.3390/ijms252211965. PMC11593382 39596036

[20] Choi J. , Le D. D. , Roh N. et al., Chemical Composition and Biological Activities of *Lagopsis supina* Extract, Pharmaceuticals. (2025) 18, no. 2, 10.3390/ph18020150. PMC11860115 40005965

[21] Le D. D. , Kim Y. R. , Yu S. , Dang T. , Lee K. T. , and Lee M. , Phytochemical and Bioactivities of Flavonoid Glycosides from *Vicia bungei* , Food Chemistry. (2024) 460, 10.1016/j.foodchem.2024.140541. 39137573

[22] Subedi L. , Le D. D. , Kim E. et al., Sulforaphane-Rich Broccoli Sprout Extract Promotes Hair Regrowth via Enhanced Dihydrotestosterone Metabolism, International Journal of Molecular Sciences. (2025) 26, no. 15, 10.3390/ijms26157467. PMC12347391 40806594

[23] Nothias L. F. , Nothias-Esposito M. , da Silva R. et al., Bioactivity-Based Molecular Networking for Drug Discovery, Journal of Natural Products. (2018) 81, no. 4, 758–767, 10.1021/acs.jnatprod.7b00737. 29498278

[24] Hurley T. D. , Bosron W. F. , Stone C. L. , and Amzel L. M. , Structures of Three Human *β* Alcohol Dehydrogenase Variants, Journal of Molecular Biology. (1994) 239, no. 3, 415–429, 10.1006/jmbi.1994.1382. 8201622

[25] Chernyshova I. , Markush P. , Zavilopulo A. , and Shpenik O. , Electron Impact Ionization of the Gas-Phase Sorbitol, European Physical Journal D: Atomic, Molecular and Optical Physics. (2015) 69, no. 3, 10.1140/epjd/e2015-50641-7.

[26] Dos Santos G. F. , Lima N. M. , Lima G. S. et al., Mass Spectrometry-Based Untargeted Metabolomics Approaches for Comprehensive Structural Annotation of Bioactive Metabolites From Bushy Cashew (*Anacardium humile*) Fruits, S Afr J Bot. (2023) 163, 121–134, 10.1016/j.sajb.2023.10.030.

[27] Scheubert K. , Hufsky F. , and Böcker S. , Computational Mass Spectrometry for Small Molecules, Journal of Cheminformatics. (2013) 5, no. 1, 10.1186/1758-2946-5-12. PMC3648359 23453222

[28] Le Pogam P. , Legouin B. , Le Lamer A. C. , Boustie J. , and Rondeau D. , Analysis of the Cyanolichen *Lichina pygmaea* Metabolites Using in Situ DART-MS, Journal of Mass Spectrometry. (2015) 50, no. 3, 454–462, 10.1002/jms.3549. 25800181

[29] Ramiro-Cortijo D. , Rebollo-Hernanz M. , Rodríguez-Rodríguez P. , Ruvira S. , Arribas S. M. , and Martín-Cabrejas M. A. , Untargeted Metabolomics and Chemometrics Elucidate Plasma Profile Changes Induced by Cocoa Shell, Nutrients. (2025) 17, no. 5, 10.3390/nu17050885. PMC11902091 40077756

[30] Dutschke J. , Suchowski M. , and Pietsch J. , Simultaneous Determination of Catechins and Pyrogallol in Deer Intoxications by HPLC-MS/MS, Journal of Chromatography B. (2021) 1180, 10.1016/j.jchromb.2021.122886. 34428648

[31] Kumar K. K. , Goodwin C. R. , Uhouse M. A. et al., Untargeted Metabolic Profiling Identifies Interactions Between Huntington’s Disease and Manganese Status, Metallomics. (2015) 7, no. 2, 363–370, 10.1039/c4mt00223g. PMC4326616 25599126

[32] Zhang Z. , Chen H. , Chan K. K. , Budd T. , and Ganapathi R. , GC-MS Analysis of Perillyl Alcohol Metabolites in Plasma, Journal of Chromatography B. (1999) 728, no. 1, 85–95, 10.1016/s0378-4347(99)00065-1. 10379660

[33] Guo B. , Yang L. , Li H. et al., Chemical Components and Metabolite Pathways in *Ephedra* Species Based on HS-GC-MS and UPLC-Q-TOF-MS, Frontiers in Plant Science. (2024) 15, 10.3389/fpls.2024.1421008. PMC11205265 38933459

[34] Shi J. , Gao X. , Zhang A. , Qin X. , and Du G. , Characterization of Multiple Components of Guilingji by UHPLC-MS and NMR, Journal of Pharmaceutical Analysis. (2021) 12, no. 3, 460–469, 10.1016/j.jpha.2021.09.013. PMC9257439 35811626

[35] Huang A. , Xu H. , Zhan R. et al., Metabolic Profile of Skimmianine in Rats, Molecules. (2017) 22, no. 4, 10.3390/molecules22040489. PMC6154341 28333075

[36] Wang X. , Hu Z. Y. , Hu Q. , Liu Z. M. , and Chen X. D. , Yigan Mingmu Decoction Treats Diabetic Retinopathy via Müller Cell Autophagy, Phytomedicine. (2026) 154, 10.1016/j.phymed.2026.158002. 41846099

[37] Lohani H. , Kumar A. , Bidarakundi V. , Agrawal L. , Haider S. Z. , and Chauhan N. K. , Identification of Fatty Acids in *Cinnamomum tamala* Using UPLC-Q-TOF-MSE, Molecules. (2024) 29, no. 16, 10.3390/molecules29163760. PMC11356901 39202838

[38] Angeloni S. , Marconi R. , Piatti D. et al., Discovery of New Molecules in White Truffle via UHPLC-QTOF-MS, Food Chemistry. (2025) 477, 10.1016/j.foodchem.2025.143562. 40015022

[39] Abukhalil M. H. , Hussein O. E. , Bin-Jumah M. et al., Farnesol Attenuates Oxidative Stress and Liver Injury in Hypercholesterolemic Rats, Environmental Science & Pollution Research. (2020) 27, 30118–30132, 10.1007/s11356-020-09296-w. 32449150

[40] Zhang W. , Wang M. , Wang W. et al., Detection of Acetaldehyde Dehydrogenase Inhibitors in Foods, Lebensmittel-Wissenschaft & Technologie. (2025) 223, 10.1016/j.lwt.2025.117768.

[41] Bello A. S. , Uzairu A. , Shallangwa G. A. , Abdulkadir I. , Mahmood A. A. R. , and Ibrahim M. T. , In Silico Identification of HCV NS5B Inhibitors, Silico Research in Biomedicine. (2025) 1, 10.1016/j.insi.2025.100104.

[42] Ma X. , Xiong S. , Xiang F. et al., Protective Effect of Phenolic Acids on Liver Disease, Antioxidants. (2025) 14, no. 10. 10.3390/antiox14101247 PMC12562122 41154556

[43] Choi J. J. , Yim S. H. , Choi J. H. , Park J. H. , Nam S. H. , and Lee H. C. , Antioxidant Activity of *Pyrus pyrifolia* Fruit in Different Cultivars and Parts, Food Science and Preservation. (2013) 20, no. 2, 222–226, 10.11002/kjfp.2013.20.2.222.

[44] Leung T. M. and Nieto N. , CYP2E1 and Oxidant Stress in Alcoholic Liver Disease, Journal of Hepatology. (2013) 58, no. 2, 395–398, 10.1016/j.jhep.2012.08.018. 22940046

[45] Schattenberg J. M. and Czaja M. J. , Regulation of CYP2E1-induced Oxidative Stress by JNK Signaling, Redox Biology. (2014) 3, 7–15, 10.1016/j.redox.2014.09.004. PMC4218941 25462060

[46] Cederbaum A. I. , Role of CYP2E1 in Ethanol-Induced Oxidant Stress and Fatty Liver, Digestive Diseases. (2011) 28, no. 6, 802–811, 10.1159/000324289. PMC3211519 21525766

[47] Feng X. M. , Xiong J. , Qin H. et al., Fluoxetine Induces Hepatic Lipid Accumulation via SREBP1c-Related Lipogenesis, CNS Neuroscience and Therapeutics. (2012) 18, no. 12, 974–980, 10.1111/cns.12014. PMC6493645 23137031

[48] Yadav U. C. and Ramana K. V. , Regulation of NF-κB Inflammatory Signaling by Lipid Peroxidation-Derived Aldehydes, Oxidative Medicine and Cellular Longevity. (2013) 2013. 10.1155/2013/690545 PMC3654319 23710287

[49] Luedde T. and Schwabe R. F. , NF-κB in the Liver: Linking Injury, Fibrosis and Hepatocellular Carcinoma, Nature Reviews Gastroenterology & Hepatology. (2011) 8, no. 2, 108–118, 10.1038/nrgastro.2010.213. PMC3295539 21293511

[50] Lin C. , Xing J. , Jiang Z. et al., Tanshinone IIA Inhibits Liver Fibrosis by Regulating COL1A1 Expression, Natural Product Communications. (2022) 17, no. 9, 10.1177/1934578X221123698.

[51] Ma H. P. , Chang H. L. , Bamodu O. A. et al., Collagen 1A1 (COL1A1) as Biomarker in Hepatocellular Carcinoma, Cancers. (2019) 11, no. 6, 10.3390/cancers11060786. PMC6627889 31181620

[52] He F. , Ru X. , and Wen T. , NRF2, a Transcription Factor for Stress Response and Beyond, International Journal of Molecular Sciences. (2020) 21, no. 13, 10.3390/ijms21134777. PMC7369905 32640524

