# Supplementary material for: Pear Peel–Enriched Residue Extract Attenuates Ethanol‐Induced Liver Injury Through Regulation of Antioxidant Signaling and Lipid Metabolism
Source: Adv Pharmacol Pharm Sci. 2026 Nov 4;2026:6147577. doi: 10.1155/adpp/6147577 (PMC13735535; doi:10.1155/adpp/6147577)
Supplement: Supplementary file 1 [file ADPP-2026-6147577-s001.docx]

**Table S1.** Identification of components from *P*. *pyrifolia* extract.

| **No.** | **Compound** | **RT (min)** | **Formula** | **Adduct** | ***m/z* (Da)** | **Error (mDa)** | **Class** |
| --- | --- | --- | --- | --- | --- | --- | --- |
| 1 | Unknown | 1.088 | - | [M+H]^+^ | 128.0192 | - | - |
| 2 | Unknown | 1.088 | - | [M+H]^+^ | 241.9994 | - | - |
| 3 | 2-Hydroxy-3,4-dimethoxybenzoic acid | 1.246 | C_9_H_10_O_5_ | [M+Na]^+^ | 221.0418 | 0.1830 | Organic acids |
| 4 | Gentiobiose | 1.285 | C_12_H_22_O_11_ | [M+K]+ | 381.0787 | 0.6410 | Terpenoids |
| 5 | D-Fructose | 1.334 | C_6_H_12_O_6_ | [M+NH_4_]^+^ | 180.0864 | 0.0004 | Glycosides |
| 6 | D-1-[(3-Carboxypropyl)amino]-1-deoxyfructose | 1.372 | C_10_H_19_NO_7_ | [M+H]^+^ | 266.1230 | 0.0030 | Alkaloids |
| 7 | D-Sorbitol | 1.420 | C_6_H_14_O_6_ | [M+H]^+^ | 183.0861 | 0.0001 | Glycosides |
| 8 | Mycosporine serinol | 1.699 | C_11_H_19_NO_6_ | [M+H]^+^ | 262.1280 | 0.0310 | Phenylpropanoids |
| 9 | Unknown | 1.699 | - | [M+H]^+^ | 280.1386 | - | - |
| 10 | Tyrosine | 1.894 | C_9_H_11_NO_3_ | [M+H]^+^ | 182.0808 | 0.3510 | Alkaloids |
| 11 | Noradrenaline | 1.952 | C_8_H_11_NO_3_ | [M+H-H_2_O]^+^ | 152.0702 | 0.3660 | Alkaloids |
| 12 | Methyl 3,4,5-trihydroxycyclohexene-1-carboxylate | 1.962 | C_8_H_12_O_5_ | [M +H_ACN]^+^ | 230.1020 | 0.2900 | Phenylpropanoids |
| 13 | Norleucine | 2.178 | C_6_H_13_NO_2_ | [M+H]^+^ | 132.1018 | 0.0760 | Alkaloids |
| 14 | Methoxyindoleacetic acid | 2.250 | C_1_1H_11_NO_3_ | [M+H]^+^ | 206.0810 | 0.9000 | Alkaloids |
| 15 | L-Prolyl-L-isoleucine | 2.379 | C_11_H_20_N_2_O_3_ | [M+H]^+^ | 229.1544 | 4.3640 | Alkaloids |
| 16 | Hydroquinone | 2.492 | C_6_H_6_O_2_ | [M+H]^+^ | 111.04394 | 0.1140 | Phenols |
| 17 | L-Tyrosine | 2.522 | C_9_H_11_NO_3_ | [M+H]^+^ | 180.0653 | 0.2440 | Alkaloids |
| 18 | N-Allyladenosine | 2.571 | - | [M+H]^+^ | 308.1338 | - | - |
| 19 | 2-Benzyl-1-methyl-1,4-dihydroquinazolin-4-one | 2.582 | C_16_H_14_N_2_O | [M+H]^+^ | 273.0963 | 0.5353 | Alkaloids |
| 20 | Unknown | 2.925 | - | [M+H]^+^ | 398.1287 | - | - |

**Table S1.** Continued

| **No.** | **Compound** | **RT (min)** | **Fomula** | **Adduct** | ***m/z* (Da)** | **Error (mDa)** | **Class** |
| --- | --- | --- | --- | --- | --- | --- | --- |
| 21 | 5,7-Dihydroxycoumarin | 3.016 | C_9_H_6_O_4_ | [M+H]^+^ | 179.0337 | 0.1980 | Phenylpropanoids |
| 22 | 2-Oxindole | 3.201 | C_8_H_7_NO | [M+H]^+^ | 134.0599 | 0.1070 | Alkaloids |
| 23 | Indole-2-carboxylic acid | 3.201 | C_9_H_7_NO_2_ | [M+NH_4_]^+^ | 179.0812 | 0.2750 | Alkaloids |
| 24 | 5-hydroxy-tryptophan | 3.250 | C_11_H_12_N_2_O_3_ | [M+H]^+^ | 219.0737 | 3.6930 | Alkaloids |
| 25 | 1-Methyladenosine | 3.624 | C_11_H_15_N_5_O_4_ | [M+H]^+^ | 282.1192 | 0.4880 | Alkaloids |
| 26 | [(2*S*,3*R*,4*S*,5*S*,6*R*)-3,4,5-trihydroxy-6-(hydroxymethyl)oxan-2-yl] (*E*)-3-phenylprop-2-enoate | 3.740 | C_15_H_18_O_7_ | [M+NH_4_]^+^ | 328.1388 | 0.2140 | Glycosides |
| 27 | Methyl 2-(4*a*,6,10*a*-trihydroxy-1-methyl-5,10-dioxo-3,4-dihydro-1H-benzo[g]isochromen-3-yl)acetate | 3.891 | C_17_H_18_O_8_ | [M+NH_4_]^+^ | 368.1336 | 0.4270 | Naphthopyranones |
| 28 | Phenylalanine | 4.038 | C_9_H_11_NO_2_ | [M+H]^+^ | 166.0861 | 0.1370 | Alkaloids |
| 29 | Indoline | 4.050 | C_8_H_9_N | [M+H]^+^ | 120.0807 | 0.0610 | Alkaloids |
| 30 | Pyrogallol | 4.431 | C_6_H_6_O_3_ | [M+H]^+^ | 127.0389 | 0.1140 | Phenols |
| 31 | N-Acetylleucine | 4.453 | C_8_H_15_NO_3_ | [M+H]^+^ | 196.0966 | 0.2469 | Fatty Acids |
| 32 | Pantothenic acid | 4.495 | C_9_H_17_NO_5_ | [M+H]^+^ | 220.1177 | 0.3200 | Alkaloids |
| 33 | 3-[2-Methoxy-3-[(2*S*, 3*R*, 4*S*, 5*S*, 6*R*)-3,4,5-trihydroxy-6-(hydroxymethyl)oxan-2-yl]oxyphenyl]propanoic acid | 4.962 | C_16_H_22_O_9_ | [M+NH_4_]^+^ | 376.1598 | 0.4580 | Glycosides |
| 34 | Piperic acid | 5.395 | C_12_H_10_O_4_ | [M+NH_4_]^+^ | 236.0916 | 0.1680 | Fatty Acids |
| 35 | Methyl Asterrate | 5.586 | C_18_H_18_O_8_ | [M+NH_4_]^+^ | 380.1337 | 0.3360 | Phenols |
| 36 | 4,7,8-Trimethoxyfuro[2,3-b]quinoline | 5.999 | C_14_H_13_NO_4_ | [M+H]^+^ | 260.0915 | 0.2440 | Alkaloids |
| 37 | 5-Phenyllevulinic acid | 6.062 | C_11_H_12_O_3_ | [M+NH_4_]^+^ | 210.1122 | 0.2440 | Organic acids |
| 38 | (*Z*)-3-[4-methoxy-2-[(2*S*,3*R*,4*S*,5*S*,6*R*)-3,4,5-trihydroxy-6-(hydroxymethyl)oxan-2-yl]oxyphenyl]prop-2-enoic acid | 6.253 | C_16_H_20_O_9_ | [M+NH_4_]^+^ | 374.1441 | 0.0009 | Glycosides |
| 39 | 4-(2-Methyl-6-oxopyran-3-yl)butanoic acid | 6.612 | C_10_H_12_O_4_ | [M+H]^+^ | 197.0807 | 0.1680 | Pyranone |

**Table S1.** Continued

| **No.** | **Compound** | **RT (min)** | **Formula** | **Adduct** | ***m/z* (Da)** | **Error (mDa)** | **Class** |
| --- | --- | --- | --- | --- | --- | --- | --- |
| 40 | (*E*)-9-(1,3-benzodioxol-5-yl)-1-piperidin-1-ylnon-8-en-1-one | 6.629 | C_21_H_29_NO_3_ | [M+H]^+^ | 344.2273 | 4.2720 | Alkaloids |
| 41 | 7-Hydroxy-coumarin | 6.746 | C_9_H_6_O_3_ | [M+H]^+^ | 163.0388 | 0.1680 | Phenylpropanoids |
| 42 | Chlorogenic acid | 6.789 | C_16_H_18_O_9_ | [M+H]^+^ | 355.1021 | 0.0011 | Polyphenols |
| 43 | (*E*)-3-[4-[(2*S*,3*R*,4*S*,5*S*,6*R*)-3,4,5-trihydroxy-6-(hydroxymethyl)oxan-2-yl]oxyphenyl]prop-2-enoic acid | 6.844 | C_15_H_18_O_8_ | [M+NH_4_]^+^ | 344.1337 | 0.0003 | Glycosides |
| 44 | 5-Aminolevulinic acid | 7.014 | C_5_H_9_NO_3_ | [M+Na]^+^ | 154.0497 | 0.0610 | Alkaloids |
| 45 | 4-Methoxy-6-prop-2-enyl-1,3-benzodioxole | 7.101 | C_11_H_12_O_3_ | [M+H]^+^ | 193.0857 | 0.1830 | Phenylpropanoids |
| 46 | Unknown | 7.154 | - | [M+H]^+^ | 388.2535 | - | - |
| 47 | 2-[(2-Amino-3-methylbutanoyl)amino]-3-phenylpropanoic acid | 7.154 | C_14_H_20_N_2_O_3_ | [M+H]^+^ | 265.1543 | 0.0003 | Alkaloids |
| 48 | Cryptotanshinone | 7.185 | C_19_H_20_O_3_ | [M+H]^+^ | 297.1443 | 0.2111 | Terpenoids |
| 49 | (*E*)-3-(3,4,5-trimethoxyphenyl)prop-2-enal | 7.196 | C_12_H_14_O_4_ | [M+H]^+^ | 223.0962 | 0.2590 | Aldehydes |
| 50 | Roseoside | 7.544 | C_19_H_30_O_8_ | [M+H]^+^ | 387.2008 | 0.0022 | Glycosides |
| 51 | Sinapoyl malate | 7.545 | C_15_H_16_O_9_ | [M+H-C_4_H_6_O_5_]^+^ | 207.1375 | 0.4270 | Organic acids |
| 52 | 4-[4-(*β*-D-glucopyranosyloxy)-2-hydroxy-2,6,6-trimethylcyclohexylidene]-3-Buten-2-one | 7.545 | C_19_H_30_O_8_ | [M+Na]^+^ | 409.1824 | 0.8540 | Terpenoids |
| 53 | (*E*)-3-(4-hydroxy-3-methoxyphenyl)prop-2-enamide | 7.826 | C_10_H_11_NO_3_ | [M+H]^+^ | 194.0810 | 0.1980 | Alkaloids |
| 54 | 8-Methoxypsoralen | 7.826 | C_12_H_8_O_4_ | [M+NH_4_]^+^ | 234.0735 | 2.5790 | Furocoumarins |
| 55 | Abieta-8(14),9(11),12-triene-7,18-diol | 7.847 | C_20_H_30_O_2_ | [M+H+ACN]^+^ | 344.2540 | 0.3852 | Diterpenoids |
| 56 | Pyroglutamylisoleucine | 8.015 | C_11_H_18_N_2_O_4_ | [M+H]^+^ | 243.1337 | 0.3200 | Dipeptides |
| 57 | 2,3,8,9,10-Pentamethoxy-6a,11a-dihydro-6H-[1]benzofuro[3,2-c]chromene | 8.036 | C_20_H_22_O_7_ | [M+H]^+^ | 375.1433 | 0.4880 | Benzofurochromene |
| 58 | (7*E*)-11-Hydroxy-3-isobutyl-4,5,8,13,13-pentamethyl-3,3a,4,6a,9,10,11,11a,14a,15-decahydro-1H-[1,3]dioxolo[9,10]oxacyclododecino[2,3-d]isoindole-1,16(2H)-dione | 8.095 | C_27_H_41_NO_6_ | [M+H]^+^ | 476.3053 | 4.6690 | Macrolide |

**Table S1.** Continued

| **No.** | **Compound** | **RT (min)** | **Formula** | **Adduct** | ***m/z* (Da)** | **Error (mDa)** | **Class** |
| --- | --- | --- | --- | --- | --- | --- | --- |
| 59 | 4'-Hydroxy-4-methoxydalbergione | 8.185 | C_16_H_14_O_4_ | [M+H]^+^ | 271.0962 | 0.2140 | Flavonoids |
| 60 | Miriquidic acid | 8.185 | C_25_H_30_O_8_ | [M+Na]^+^ | 481.1851 | 1.0990 | Organic acids |
| 61 | Selaginpulvilin T | 8.185 | C_36_H_28_O_5_ | [M+H]^+^ | 539.1882 | 1.1600 | Polyalcohols |
| 62 | 3-Phenyllactic acid | 8.369 | C_9_H_10_O_3_ | [M+H]^+^ | 167.0702 | 0.1070 | Organic acids |
| 63 | 2-[(1*R*,2*R*)-3-oxo-2-[(*Z*)-5-[3,4,5-trihydroxy-6-(hydroxymethyl)oxan-2-yl]oxypent-2-enyl]cyclopentyl]acetic acid | 8.567 | C_18_H_28_O_9_ | [M+H]^+^ | 389.1780 | 2.6550 | Fatty acyl glycosides of mono- and disaccharides |
| 64 | Norcocaine | 8.580 | C_16_H_19_NO_4_ | [M+H]^+^ | 290.1384 | 0.3660 | Alkaloids |
| 65 | 3,5-Dihydroxydecanoic acid | 8.591 | C_10_H_20_O_4_ | [M+H]^+^ | 205.1433 | 0.1070 | Fatty acids |
| 66 | Indole-3-pyruvic acid | 8.649 | C_11_H_9_NO_3_ | [M+H]^+^ | 204.0654 | 0.1370 | Alkaloids |
| 67 | Argaminolic A | 8.649 | C_11_H_11_NO_4_ | [M+H]^+^ | 222.0759 | 0.0760 | Amino phenolics |
| 68 | Sweroside | 8.717 | C_16_H_22_O_9_ | [M+Na]^+^ | 381.1175 | 1.9230 | O-glycosyl compounds |
| 69 | 4-(Hydroxymethyl)-1-isopropyl-3-cyclohexen-1-yl beta-D-glucopyranoside | 8.728 | C_16_H_28_O_7_ | [M+Na]^+^ | 355.1724 | 0.3610 | Terpenoids |
| 70 | 4-(Beta-D-glucopyranosyloxy)-6-methoxy-5-Benzofuranpropanoic acid | 8.728 | C_18_H_22_O_10_ | [M+Na]^+^ | 421.1099 | 0.5800 | Benzofurans |
| 71 | (6*E*)-14-hydroxy-4,14,16-trimethyl-8-methylidene-17-tridecyl-1-oxa-4,9,12-triazacycloheptadec-6-ene-2,5,10,13,15-pentone | 8.816 | C_30_H_49_N_3_O_7_ | [M+H]^+^ | 564.3583 | 1.6480 | Alkaloids |
| 72 | Cinnamic acid | 8.843 | C_9_H_8_O_2_ | [M+H]^+^ | 149.0596 | 0.4120 | Cinnamates |
| 73 | 2-Phenylethyl-galactoside | 8.878 | C_14_H_20_O_6_ | [M+NH_4_]^+^ | 302.1596 | 0.0004 | Glycosides |
| 74 | Unknown | 8.893 | - | [M+H]^+^ | 338.1806 | - | - |
| 75 | Coniferin | 8.893 | C_16_H_22_O_8_ | [M+H]^+^ | 343.1362 | 2.5330 | Cinnamates |
| 76 | 8-Hydroxypinoresinol-4'-*O*-*β*-D-Glucopyranoside | 9.114 | C_26_H_32_O_12_ | [M+NH_4_]^+^ | 554.2226 | 0.0004 | Glycosides |

**Table S1.** Continued

| **No.** | **Compound** | **RT (min)** | **Formula** | **Adduct** | ***m/z* (Da)** | **Error (mDa)** | **Class** |
| --- | --- | --- | --- | --- | --- | --- | --- |
| 77 | Hydroxyphenyllactate | 9.310 | C_11_H_8_O_4_ | [M+Na]^+^ | 205.0493 | 0.2493 | Ketones |
| 78 | 8-Hydroxy-3-methyl-1-oxo-3,4-dihydroisochromene-5-carboxylic acid | 9.310 | C_11_H_10_O_5_ | [M+H]^+^ | 223.0598 | 0.2750 | Phenylpropanoids |
| 79 | 4-(Hydroxymethyl)-1-isopropyl-3-cyclohexen-1-yl *β*-D-glucopyranoside | 9.310 | C_16_H_28_O_7_ | [M+Na]^+^ | 355.1723 | 0.3970 | Glycosides |
| 80 | 2-(3,4-Dihydroxyphenyl)-5-hydroxy-7-[(2*S*,3*R*,4*S*,5*S*,6*R*)-3,4,5-trihydroxy-6-[[(2*R*,3*R*,4*R*,5*R*,6*S*)-3,4,5-trihydroxy-6-methyloxan-2-yl]oxymethyl]oxan-2-yl]oxychromen-4-one | 9.448 | C_27_H_30_O_15_ | [M+H]^+^ | 595.1654 | 0.0004 | Flavonoids |
| 81 | Villol | 9.668 | C_23_H_22_O_9_ | [M+NH_4_]^+^ | 460.1596 | 0.5490 | Flavonoids |
| 82 | {(1*R*,2*R*)-2-[(2*Z*)-5-Hydroxy-2-penten-1-yl]-3-oxocyclopentyl}acetic acid | 9.704 | C_13_H_14_N_4_ | [M+Na]^+^ | 249.1118 | 2.0900 | Alkaloids |
| 83 | Unknown | 9.916 | - | [M+H]^+^ | 552.2068 | - | - |
| 84 | (6*S*,7*aR*)-5,6,7,7*a*-tetrahydro-6-hydroxy-4,4,7*a*-trimethyl-2(4H)-Benzofuranone | 10.176 | C_11_H_16_O_3_ | [M+Na]^+^ | 219.1014 | 2.2430 | Benzofurans |
| 85 | Perillyl alcohol | 10.432 | C_10_H_16_O | [M+H]^+^ | 153.1272 | 0.2290 | Terpenoids |
| 86 | Neodiosmin | 10.606 | C_28_H_32_O_15_ | [M+H]^+^ | 609.1808 | 0.0032 | Flavonoids |
| 87 | 8-(2-Hydroxypropan-2-yl)furo[2,3-h]chromen-2-one | 10.793 | C_14_H_12_O_4_ | [M+H]^+^ | 245.0806 | 0.2590 | Coumarins |
| 88 | 5,7-Dihydroxy-2-(4-hydroxy-3-methoxyphenyl)-3-[3,4,5-trihydroxy-6-(hydroxymethyl)oxan-2-yl]oxychromen-4-one | 11.017 | C_22_H_22_O_12_ | [M+H]^+^ | 479.1177 | 0.0003 | Glycosides |
| 89 | 4,5-Dihydro-2',3'-epoxy-15-deoxygoyazensolide | 11.401 | C_19_H_22_O_8_ | [M+Na]^+^ | 401.1204 | 0.3050 | Terpenoids |
| 90 | [4,8-Dihydroxy-3-(3-methoxy-3-oxoprop-1-en-2-yl)-8a-methyl-5-oxo-1,2,3,4,4a,6,7,8-octahydronaphthalen-2-yl] 3,4-dihydroxy-2-methylidenebutanoate | 11.808 | C_20_H_28_O_9_ | [M+Na]^+^ | 435.1644 | 1.5870 | Organic acids |
| 91 | 3-Indoleacrylic acid | 12.141 | C_11_H_9_NO_2_ | [M+H]^+^ | 188.0705 | 0.0920 | Organic acids |
| 92 | 2-Methoxy-1,4-naphthoquinone | 12.270 | C_11_H_8_O_3_ | [M+H]^+^ | 189.0544 | 0.1830 | Naphthoquinones |
| 93 | Abscisic acid | 12.594 | C_15_H_20_O_4_ | [M+H-H_2_O]^+^ | 247.1325 | 0.0005 | Organic acids |

**Table S1.** Continued

| **No.** | **Compound** | **RT (min)** | **Formula** | **Adduct** | ***m/z* (Da)** | **Error (mDa)** | **Class** |
| --- | --- | --- | --- | --- | --- | --- | --- |
| 94 | Unknown | 12.678 | C_14_H_22_N_6_O_6_ | [M+H]^+^ | 371.1673 | 0.0920 | Alkaloids |
| 95 | (3a*R*,6*R*,7a*R*)-6-methyl-3-methylidene-6-(4-oxopentyl)-3a,4,7,7a-tetrahydro-1-benzofuran-2,5-dione | 13.484 | C_15_H_20_O_4_ | [2M+Na]^+^ | 551.2609 | 2.1360 | Terpenoids |
| 96 | 9_10-*α*-Epoxy-eremanthin | 13.496 | C_15_H_18_O_3_ | [M+H]^+^ | 247.1326 | 0.2750 | Gamma butyrolactones |
| 97 | (4*R*)- dihydro-3-methylene-4-[(2E)-3-methyl-4-(tetrahydro-4-methyl-5-oxo-2-furanyl)-2-buten-1-yl]-2(3H)-Furanone | 13.496 | C_15_H_20_O_4_ | [M+Na]^+^ | 287.1250 | 0.3360 | Terpenoids |
| 98 | 3-(Hexopyranosyloxy)-2-[(2*Z*)-2-penten-1-yl]-cyclopentaneacetic acid | 13.699 | C_18_H_30_O_8_ | [M+Na]^+^ | 397.1829 | 0.3660 | Glycosides |
| 99 | 3,4-Dihydro-6-hydroxy-8-methoxy-3-methyl-1H-2-Benzopyran-1-one | 14.325 | C_11_H_12_O_4_ | [M+H]^+^ | 209.0806 | 0.2290 | Benzopyrans |
| 100 | Neobavaisoflavone | 14.325 | C_20_H_18_O_4_ | [2M+Na]^+^ | 667.2358 | 5.8590 | Flavonoids |
| 101 | Syringaresinol | 14.612 | C_22_H_26_O_8_ | [M+H]^+^ | 417.1538 | 1.1900 | Polyphenols |
| 102 | Unknown | 14.612 | - | [M+H]^+^ | 637.2249 | - | - |
| 103 | Unknown | 14.851 | - | [M+H]^+^ | 429.2817 | - | - |
| 104 | Sparteine-15-carboxylic acid | 14.875 | C_16_H_26_N_2_O_2_ | [M+H]^+^ | 279.2040 | 2.9600 | Quinolizines |
| 105 | Unknown | 14.875 | - | [M+H]^+^ | 257.2221 | - | - |
| 106 | 9-Oxootre | 15.465 | C_18_H_28_O_3_ | [M+H-H_2_O]^+^ | 275.2003 | 0.0003 | Fatty acids |
| 107 | (10*E*,15*E*)-9,12,13-trihydroxyoctadeca-10,15-dienoic acid | 15.478 | C_18_H_32_O_5_ | [M+H]^+^ | 351.2140 | 0.0310 | Fatty acids |
| 108 | 9-Methoxycarbonyldec-9-enoic acid | 15.568 | C_12_H_20_O_4_ | [M+H]^+^ | 229.1432 | 0.2750 | Fatty acids |
| 109 | Unknown | 15.574 | - | [M+H]^+^ | 211.0751 | - | - |
| 110 | Dipropyl phthalate | 15.585 | C_14_H_18_O_4_ | [M+H]^+^ | 251.1251 | 0.2730 | Organic acids |
| 111 | Unknown | 15.609 | - | [M+H]^+^ | 607.2146 | - | - |
| 112 | 9(10)-EpOME | 16.165 | C_18_H_32_O_3_ | [M+H-H_2_O]^+^ | 279.2316 | 0.0004 | Fatty acids |

**Table S1.** Continued

| **No.** | **Compound** | **RT (min)** | **Formula** | **Adduct** | ***m/z* (Da)** | **Error (mDa)** | **Class** |
| --- | --- | --- | --- | --- | --- | --- | --- |
| 113 | 8-Hydroxy-8-(3-octyloxiran-2-yl)octanoic acid | 16.165 | C_18_H_3_4O_4_ | [M+H-H_2_O]^+^ | 297.2422 | 0.1830 | Fatty acids |
| 114 | 5-[5-Hydroxy-3-(hydroxymethyl)pentyl]-8a-(hydroxymethyl)-5,6-dimethyl-3,4,4a,6,7,8-hexahydronaphthalene-1-carboxylic acid | 16.165 | C_20_H_34_O_5_ | [M+H]^+^ | 355.2453 | 2.5940 | Terpenoids |
| 115 | (*Z*)-9,12,13-trihydroxyoctadec-15-enoic acid | 16.514 | C_18_H_34_O_5_ | [M+NH_4_]^+^ | 348.2741 | 1.3430 | Fatty acids |
| 116 | FA 18:3+1O | 16.525 | C_18_H_30_O_3_ | [M+H]^+^ | 295.2264 | 2.0450 | Fatty acids |
| 117 | (9*Z*)-5,8,11-Trihydroxyoctadec-9-enoic acid | 16.525 | C_18_H_34_O_5_ | [M+Na]^+^ | 353.2294 | 1.7910 | Fatty acids |
| 118 | Lauryldiethanolamine | 19.443 | C_16_H_35_NO_2_ | [M+H]^+^ | 274.2738 | 0.5190 | Alkaloids |
| 119 | Gelomulide N | 21.540 | C_24_H_32_O_7_ | [M+H-H_2_O]^+^ | 415.2109 | 0.6100 | Naphthofurans |
| 120 | 7b,9-Dihydroxy-3-(hydroxymethyl)-1,1,6,8-tetramethyl-5-oxo-1,1a,1b,4,4a,5,7a,7b,8,9-decahydro-9aH-cyclopropa[3,4]benzo[1,2-e]azulen-9a-yl acetate | 21.540 | C_22_H_30_O_6_ | [M+H+ACN]^+^ | 432.2375 | 0.4270 | Terpenoids |

**Table S2.** Experimental and reference spectra of compound annotations.

| **No.** | **Compound** | **RT (min)** | **Formula** | **M/Z** | **Error (mDa)** | **Note** |
| --- | --- | --- | --- | --- | --- | --- |
| 1 | Unknown | 1.088 | - | 128.0192 | - |  |
| 2 | Unknown | 1.088 | - | 241.9994 | - |  |
| 3 | 2-hydroxy-3,4-dimethoxybenzoic acid | 1.246 | C_9_H_10_O_5_ | 221.0418 | 0.1830 |  |
| 4 | Gentiobiose | 1.285 | C_12_H_22_O_11_ | 381.0787 | 0.6410 |  |
| 5 | D-Fructose | 1.334 | C_6_H_12_O_6_ | 180.0864 | 0.0004 |  |
| 6 | D-1-[(3-Carboxypropyl)amino]-1-deoxyfructose | 1.372 | C_10_H_19_NO_7_ | 266.1230 | 0.0030 |  |
| 7 | D-Sorbitol | 1.420 | C_6_H_14_O_6_ | 183.0861 | 0.0001 |  |
| 8 | Mycosporine serinol | 1.699 | C_11_H_19_NO_6_ | 262.1280 | 0.0310 |  |
| 9 | Unknown | 1.699 | - | 280.1386 | - |  |
| 10 | Tyrosine | 1.894 | C_9_H_11_NO_3_ | 182.0808 | 0.3510 |  |
| 11 | Noradrenaline | 1.952 | C_8_H_11_NO_3_ | 152.0702 | 0.3660 |  |
| 12 | Methyl 3,4,5-trihydroxycyclohexene-1-carboxylate | 1.962 | C_8_H_12_O_5_ | 230.1020 | 0.2900 |  |
| 13 | Norleucine | 2.178 | C_6_H_13_NO_2_ | 132.1018 | 0.0760 |  |
| 14 | Methoxyindoleacetic acid | 2.250 | C_1_1H_11_NO_3_ | 206.0810 | 0.9000 |  |
| 15 | L-Prolyl-L-isoleucine | 2.379 | C_11_H_20_N_2_O_3_ | 229.1544 | 4.3640 |  |
| 16 | Hydroquinone | 2.492 | C_6_H_6_O_2_ | 111.04394 | 0.1140 |  |
| 17 | L-Tyrosine | 2.522 | C9H_11_NO_3_ | 180.0653 | 0.2440 |  |
| 18 | N-Allyladenosine | 2.571 | - | 308.1338 | - |  |
| 19 | 2-benzyl-1-methyl-1,4-dihydroquinazolin-4-one | 2.582 | C_16_H_14_N_2_O | 273.0963 | 0.5353 |  |
| 20 | Unknown | 2.925 | - | 398.1287 | - |  |
| 21 | 5,7-dihydroxycoumarin | 3.016 | C_9_H_6_O_4_ | 179.0337 | 0.1980 |  |
| 22 | 2-oxindole | 3.201 | C_8_H_7_NO | 134.0599 | 0.1070 |  |
| 23 | Indole-2-carboxylic acid | 3.201 | C_9_H_7_NO_2_ | 179.0812 | 0.2750 |  |
| 24 | 5-hydroxy-tryptophan | 3.250 | C_11_H_12_N_2_O_3_ | 219.0737 | 3.6930 |  |
| 25 | 1-methyladenosine | 3.624 | C_11_H_15_N_5_O_4_ | 282.1192 | 0.4880 |  |
| 26 | [(2*S*,3*R*,4*S*,5*S*,6*R*)-3,4,5-trihydroxy-6-(hydroxymethyl)oxan-2-yl] (*E*)-3-phenylprop-2-enoate | 3.740 | C_15_H_18_O_7_ | 328.1388 | 0.2140 |  |
| 27 | Methyl 2-(4a,6,10a-trihydroxy-1-methyl-5,10-dioxo-3,4-dihydro-1H-benzo[g]isochromen-3-yl)acetate | 3.891 | C_17_H_18_O_8_ | 368.1336 | 0.4270 |  |
| 28 | Phenylalanine | 4.038 | C_9_H_11_NO_2_ | 166.0861 | 0.1370 |  |
| 29 | Indoline | 4.050 | C_8_H_9_N | 120.0807 | 0.0610 |  |
| 30 | Pyrogallol | 4.431 | C_6_H_6_O_3_ | 127.0389 | 0.1140 |  |
| 31 | N-Acetylleucine | 4.453 | C_8_H_15_NO_3_ | 196.0966 | 0.2469 |  |
| 32 | Pantothenic acid | 4.495 | C_9_H_17_NO_5_ | 220.1177 | 0.3200 |  |
| 33 | 3-[2-methoxy-3-[(2*S*, 3*R*, 4*S*, 5*S*, 6*R*)-3,4,5-trihydroxy-6-(hydroxymethyl)oxan-2-yl]oxyphenyl]propanoic acid | 4.962 | C_16_H_22_O_9_ | 376.1598 | 0.4580 |  |
| 34 | Piperic acid | 5.395 | C_12_H_10_O_4_ | 236.0916 | 0.1680 |  |
| 35 | Methyl Asterrate | 5.586 | C_18_H_18_O_8_ | 380.1337 | 0.3360 |  |
| 36 | 4,7,8-Trimethoxyfuro[2,3-b]quinoline | 5.999 | C_14_H_13_NO_4_ | 260.0915 | 0.2440 |  |
| 37 | 5-Phenyllevulinic acid | 6.062 | C_11_H_12_O_3_ | 210.1122 | 0.2440 |  |
| 38 | (*Z*)-3-[4-methoxy-2-[(2*S*,3*R*,4*S*,5*S*,6*R*)-3,4,5-trihydroxy-6-(hydroxymethyl)oxan-2-yl]oxyphenyl]prop-2-enoic acid | 6.253 | C_16_H_20_O_9_ | 374.1441 | 0.0009 |  |
| 39 | 4-(2-methyl-6-oxopyran-3-yl)butanoic acid | 6.612 | C_10_H_12_O_4_ | 197.0807 | 0.1680 |  |
| 40 | (*E*)-9-(1,3-benzodioxol-5-yl)-1-piperidin-1-ylnon-8-en-1-one | 6.629 | C_21_H_29_NO_3_ | 344.2273 | 4.2720 |  |
| 41 | 7-hydroxy-coumarin | 6.746 | C_9_H_6_O_3_ | 163.0388 | 0.1680 |  |
| 42 | Chlorogenic acid | 6.789 | C_16_H_18_O_9_ | 355.1021 | 0.0011 |  |
| 43 | (*E*)-3-[4-[(2*S*,3*R*,4*S*,5*S*,6*R*)-3,4,5-trihydroxy-6-(hydroxymethyl)oxan-2-yl]oxyphenyl]prop-2-enoic acid | 6.844 | C_15_H_18_O_8_ | 344.1337 | 0.0003 |  |
| 44 | 5-aminolevulinic acid | 7.014 | C_5_H_9_NO_3_ | 154.0497 | 0.0610 |  |
| 45 | 4-methoxy-6-prop-2-enyl-1,3-benzodioxole | 7.101 | C_11_H_12_O_3_ | 193.0857 | 0.1830 |  |
| 46 | Unknown | 7.154 | - | 388.2535 | - |  |
| 47 | 2-[(2-amino-3-methylbutanoyl)amino]-3-phenylpropanoic acid | 7.154 | C_14_H_20_N_2_O_3_ | 265.1543 | 0.0003 |  |
| 48 | Cryptotanshinone | 7.185 | C_19_H_20_O_3_ | 297.1443 | 0.2111 |  |
| 49 | (*E*)-3-(3,4,5-trimethoxyphenyl)prop-2-enal | 7.196 | C_12_H_14_O_4_ | 223.0962 | 0.2590 |  |
| 50 | Roseoside | 7.544 | C_19_H_30_O_8_ | 387.2008 | 0.0022 |  |
| 51 | Sinapoyl malate | 7.545 | C_15_H_16_O_9_ | 207.1375 | 0.4270 |  |
| 52 | 4-[4-(*β*-D-glucopyranosyloxy)-2-hydroxy-2,6,6-trimethylcyclohexylidene]-3-Buten-2-one | 7.545 | C_19_H_30_O_8_ | 409.1824 | 0.8540 |  |
| 53 | (*E*)-3-(4-hydroxy-3-methoxyphenyl)prop-2-enamide | 7.826 | C_10_H_11_NO_3_ | 194.0810 | 0.1980 |  |
| 54 | 8-Methoxypsoralen | 7.826 | C_12_H_8_O_4_ | 234.0735 | 2.5790 |  |
| 55 | Abieta-8(14),9(11),12-triene-7,18-diol | 7.847 | C_20_H_30_O_2_ | 344.2540 | 0.3852 |  |
| 56 | Pyroglutamylisoleucine | 8.015 | C_11_H_18_N_2_O_4_ | 243.1337 | 0.3200 |  |
| 57 | 2,3,8,9,10-pentamethoxy-6a,11a-dihydro-6H-[1]benzofuro[3,2-c]chromene | 8.036 | C_20_H_22_O_7_ | 375.1433 | 0.4880 |  |
| 58 | (7*E*)-11-Hydroxy-3-isobutyl-4,5,8,13,13-pentamethyl-3,3a,4,6a,9,10,11,11a,14a,15-decahydro-1H-[1,3]dioxolo[9,10]oxacyclododecino[2,3-d]isoindole-1,16(2H)-dione | 8.095 | C_27_H_41_NO_6_ | 476.3053 | 4.6690 |  |
| 59 | 4'-Hydroxy-4-methoxydalbergione | 8.185 | C_16_H_14_O_4_ | 271.0962 | 0.2140 |  |
| 60 | Miriquidic acid | 8.185 | C_25_H_30_O_8_ | 481.1851 | 1.0990 |  |
| 61 | Selaginpulvilin T | 8.185 | C_36_H_28_O_5_ | 539.1882 | 1.1600 |  |
| 62 | 3-phenyllactic acid | 8.369 | C_9_H_10_O_3_ | 167.0702 | 0.1070 |  |
| 63 | 2-[(1R,2R)-3-oxo-2-[(Z)-5-[3,4,5-trihydroxy-6-(hydroxymethyl)oxan-2-yl]oxypent-2-enyl]cyclopentyl]acetic acid | 8.567 | C_18_H_28_O_9_ | 389.1780 | 2.6550 |  |
| 64 | Norcocaine | 8.580 | C_16_H_19_NO_4_ | 290.1384 | 0.3660 |  |
| 65 | 3,5-dihydroxydecanoic acid | 8.591 | C_10_H_20_O_4_ | 205.1433 | 0.1070 |  |
| 66 | Indole-3-pyruvic acid | 8.649 | C_11_H_9_NO_3_ | 204.0654 | 0.1370 |  |
| 67 | Argaminolic A | 8.649 | C_11_H_11_NO_4_ | 222.0759 | 0.0760 |  |
| 68 | Sweroside | 8.717 | C_16_H_22_O_9_ | 381.1175 | 1.9230 |  |
| 69 | 4-(Hydroxymethyl)-1-isopropyl-3-cyclohexen-1-yl beta-D-glucopyranoside | 8.728 | C_16_H_28_O_7_ | 355.1724 | 0.3610 |  |
| 70 | 4-(beta-D-glucopyranosyloxy)-6-methoxy-5-Benzofuranpropanoic acid | 8.728 | C_18_H_22_O_10_ | 421.1099 | 0.5800 |  |
| 71 | (6*E*)-14-hydroxy-4,14,16-trimethyl-8-methylidene-17-tridecyl-1-oxa-4,9,12-triazacycloheptadec-6-ene-2,5,10,13,15-pentone | 8.816 | C_30_H_49_N_3_O_7_ | 564.3583 | 1.6480 |  |
| 72 | Cinnamic acid | 8.843 | C_9_H_8_O_2_ | 149.0596 | 0.4120 |  |
| 73 | 2-Phenylethyl-galactoside | 8.878 | C_14_H_20_O_6_ | 302.1596 | 0.0004 |  |
| 74 | Unknown | 8.893 | - | 338.1806 | - |  |
| 75 | Coniferin | 8.893 | C_16_H_22_O_8_ | 343.1362 | 2.5330 |  |
| 76 | 8-Hydroxypinoresinol-4'-*O*-*β*-D-Glucopyranoside | 9.114 | C_26_H_32_O_12_ | 554.2226 | 0.0004 |  |
| 77 | Hydroxyphenyllactate | 9.310 | C_11_H_8_O_4_ | 205.0493 | 0.2493 |  |
| 78 | 8-hydroxy-3-methyl-1-oxo-3,4-dihydroisochromene-5-carboxylic acid | 9.310 | C_11_H_10_O_5_ | 223.0598 | 0.2750 |  |
| 79 | 4-(Hydroxymethyl)-1-isopropyl-3-cyclohexen-1-yl *β*-D-glucopyranoside | 9.310 | C_16_H_28_O_7_ | 355.1723 | 0.3970 |  |
| 80 | 2-(3,4-dihydroxyphenyl)-5-hydroxy-7-[(2*S*,3*R*,4*S*,5*S*,6*R*)-3,4,5-trihydroxy-6-[[(2*R*,3*R*,4*R*,5*R*,6*S*)-3,4,5-trihydroxy-6-methyloxan-2-yl]oxymethyl]oxan-2-yl]oxychromen-4-one | 9.448 | C_27_H_30_O_15_ | 595.1654 | 0.0004 |  |
| 81 | Villol | 9.668 | C_23_H_22_O_9_ | 460.1596 | 0.5490 |  |
| 82 | {(1*R*,2*R*)-2-[(2*Z*)-5-Hydroxy-2-penten-1-yl]-3-oxocyclopentyl}acetic acid | 9.704 | C_13_H_14_N_4_ | 249.1118 | 2.0900 |  |
| 83 | Unknown | 9.916 | - | 552.2068 | - |  |
| 84 | (6*S*,7a*R*)-5,6,7,7a-tetrahydro-6-hydroxy-4,4,7a-trimethyl-2(4H)-Benzofuranone | 10.176 | C_11_H_16_O_3_ | 219.1014 | 2.2430 |  |
| 85 | Perillyl alcohol | 10.432 | C_10_H_16_O | 153.1272 | 0.2290 |  |
| 86 | Neodiosmin | 10.606 | C_28_H_32_O_15_ | 609.1808 | 0.0032 |  |
| 87 | 8-(2-hydroxypropan-2-yl)furo[2,3-h]chromen-2-one | 10.793 | C_14_H_12_O_4_ | 245.0806 | 0.2590 |  |
| 88 | 5,7-dihydroxy-2-(4-hydroxy-3-methoxyphenyl)-3-[3,4,5-trihydroxy-6-(hydroxymethyl)oxan-2-yl]oxychromen-4-one | 11.017 | C_22_H_22_O_12_ | 479.1177 | 0.0003 |  |
| 89 | 4,5-dihydro-2',3'-epoxy-15-deoxygoyazensolide | 11.401 | C_19_H_22_O_8_ | 401.1204 | 0.3050 |  |
| 90 | [4,8-Dihydroxy-3-(3-methoxy-3-oxoprop-1-en-2-yl)-8a-methyl-5-oxo-1,2,3,4,4a,6,7,8-octahydronaphthalen-2-yl] 3,4-dihydroxy-2-methylidenebutanoate | 11.808 | C_20_H_28_O_9_ | 435.1644 | 1.5870 |  |
| 91 | 3-Indoleacrylic acid | 12.141 | C_11_H_9_NO_2_ | 188.0705 | 0.0920 |  |
| 92 | 2-methoxy-1,4-naphthoquinone | 12.270 | C_11_H_8_O_3_ | 189.0544 | 0.1830 |  |
| 93 | Abscisic acid | 12.594 | C_15_H_20_O_4_ | 247.1325 | 0.0005 |  |
| 94 | Unknown | 12.678 | C_14_H_22_N_6_O_6_ | 371.1673 | 0.0920 |  |
| 95 | (3a*R*,6*R*,7a*R*)-6-methyl-3-methylidene-6-(4-oxopentyl)-3a,4,7,7a-tetrahydro-1-benzofuran-2,5-dione | 13.484 | C_15_H_20_O_4_ | 551.2609 | 2.1360 |  |
| 96 | 9_10-*α*-Epoxy-eremanthin | 13.496 | C_15_H_18_O_3_ | 247.1326 | 0.2750 |  |
| 97 | (4*R*)- dihydro-3-methylene-4-[(2E)-3-methyl-4-(tetrahydro-4-methyl-5-oxo-2-furanyl)-2-buten-1-yl]-2(3H)-Furanone | 13.496 | C_15_H_20_O_4_ | 287.1250 | 0.3360 |  |
| 98 | 3-(hexopyranosyloxy)-2-[(2*Z*)-2-penten-1-yl]-Cyclopentaneacetic acid | 13.699 | C_18_H_30_O_8_ | 397.1829 | 0.3660 |  |
| 99 | 3,4-Dihydro-6-hydroxy-8-methoxy-3-methyl-1H-2-Benzopyran-1-one | 14.325 | C_11_H_12_O_4_ | 209.0806 | 0.2290 |  |
| 100 | Neobavaisoflavone | 14.325 | C_20_H_18_O_4_ | 667.2358 | 5.8590 |  |
| 101 | Syringaresinol | 14.612 | C_22_H_26_O_8_ | 417.1538 | 1.1900 |  |
| 102 | Unknown | 14.612 | - | 637.2249 | - |  |
| 103 | Unknown | 14.851 | - | 429.2817 | - |  |
| 104 | Sparteine-15-carboxylic acid | 14.875 | C_16_H_26_N_2_O_2_ | 279.2040 | 2.9600 |  |
| 105 | Unknown | 14.875 | - | 257.2221 | - |  |
| 106 | 9-Oxootre | 15.465 | C_18_H_28_O_3_ | 275.2003 | 0.0003 |  |
| 107 | (10*E*,15*E*)-9,12,13-trihydroxyoctadeca-10,15-dienoic acid | 15.478 | C_18_H_32_O_5_ | 351.2140 | 0.0310 |  |
| 108 | 9-methoxycarbonyldec-9-enoic acid | 15.568 | C_12_H_20_O_4_ | 229.1432 | 0.2750 |  |
| 109 | Unknown | 15.574 | - | 211.0751 | - |  |
| 110 | Dipropyl phthalate | 15.585 | C_14_H_18_O_4_ | 251.1251 | 0.2730 |  |
| 111 | Unknown | 15.609 | - | 607.2146 | - |  |
| 112 | 9(10)-EpOME | 16.165 | C_18_H_32_O_3_ | 279.2316 | 0.0004 |  |
| 113 | 8-hydroxy-8-(3-octyloxiran-2-yl)octanoic acid | 16.165 | C_18_H_3_4O_4_ | 297.2422 | 0.1830 |  |
| 114 | 5-[5-hydroxy-3-(hydroxymethyl)pentyl]-8a-(hydroxymethyl)-5,6-dimethyl-3,4,4a,6,7,8-hexahydronaphthalene-1-carboxylic acid | 16.165 | C_20_H_34_O_5_ | 355.2453 | 2.5940 |  |
| 115 | (*Z*)-9,12,13-trihydroxyoctadec-15-enoic acid | 16.514 | C_18_H_34_O_5_ | 348.2741 | 1.3430 |  |
| 116 | FA 18:3+1O | 16.525 | C_18_H_30_O_3_ | 295.2264 | 2.0450 |  |
| 117 | (9*Z*)-5,8,11-Trihydroxyoctadec-9-enoic acid | 16.525 | C_18_H_34_O_5_ | 353.2294 | 1.7910 |  |
| 118 | Lauryldiethanolamine | 19.443 | C_16_H_35_NO_2_ | 274.2738 | 0.5190 |  |
| 119 | Gelomulide N | 21.540 | C_24_H_32_O_7_ | 415.2109 | 0.6100 |  |
| 120 | 7b,9-Dihydroxy-3-(hydroxymethyl)-1,1,6,8-tetramethyl-5-oxo-1,1a,1b,4,4a,5,7a,7b,8,9-decahydro-9aH-cyclopropa[3,4]benzo[1,2-e]azulen-9a-yl acetate | 21.540 | C_22_H_30_O_6_ | 432.2375 | 0.4270 |  |

**Table S3.** Tentative identification of major chemical constituents of *P. pyrifolia* extract in the positive polarity.

| No. | Compound | RT (min) | Formula | Adduct | *m/z* (Da) | MS^n^ |
| --- | --- | --- | --- | --- | --- | --- |
| 3 | 2-Hydroxy-3,4-dimethoxybenzoic acid | 1.246 | C_9_H_10_O_5_ | [M+Na]^+^ | 221.0418 | 221.0420/205.0557 |
| 4 | Gentiobiose | 1.285 | C_12_H_22_O_11_ | [M+K]^+^ | 381.0787 | 381.0787/342.1391 |
| 6 | D-1-[(3-carboxypropyl)amino]-1-deoxyfructose | 1.372 | C_10_H_19_NO_7_ | [M+H]^+^ | 266.1230 | 266.1230/248.1224/230.1020/146.0652 |
| 7 | D-Sorbitol | 1.420 | C_6_H_14_O_6_ | [M+H]^+^ | 183.0861 | 183.0861/147.0649/133.0606 |
| 8 | Mycosporine serinol | 1.699 | C_11_H_19_NO_6_ | [M+H]^+^ | 262.1280 | 262.1280/244.1174 |
| 10 | Tyrosine | 1.894 | C_9_H_11_NO_3_ | [M+H]^+^ | 182.0808 | 182.0809/136.0755/119.0853 |
| 12 | Methyl 3,4,5-trihydroxycyclohexene-1-carboxylate | 1.962 | C_8_H_12_O_5_ | [M+ACN+H]^+^ | 230.10201 | 230.1022/212.0916 |
| 11 | Noradrenaline | 1.952 | C_8_H_11_NO_3_ | [M+H-H_2_O]^+^ | 152.0702 | 152.0704/136.0756 |
| 13 | Norleucine | 2.178 | C_6_H_13_NO_2_ | [M+H]^+^ | 132.1018 | 132.1017/86.0964 |
| 14 | Methoxyindoleacetic acid | 2.250 | C_1_1H_11_NO_3_ | [M+H]^+^ | 206.0810 | 206.0809/146.0600 |
| 26 | [(2*S*,3*R*,4*S*,5*S*,6*R*)-3,4,5-trihydroxy-6-(hydroxymethyl)oxan-2-yl] (*E*)-3-phenylprop-2-enoate | 3.740 | C_15_H_18_O_7_ | [M+NH_4_]^+^ | 328.13885 | 328.1388/165.0757 |
| 30 | Pyrogallol | 4.431 | C_6_H_6_O_3_ | [M+H]^+^ | 127.03886 | 127.0389/109.0284/81.0335 |
| 31 | N-acetylleucine | 4.453 | C_8_H_15_NO_3_ | [M+Na]^+^ | 196.09657 | 196.0966/183.0861/180.0896/147.0650 |
| 32 | Pantothenic acid | 4.495 | C_9_H_17_NO_5_ | [M+H]^+^ | 220.11763 | 220.1178/202.1071 |
| 35 | Methyl asterrate | 5.586 | C_18_H_18_O_8_ | [M+NH_4_]^+^ | 380.13367 | 380.1336/262.1071 |
| 36 | 4,7,8-Trimethoxyfuro[2,3-b]quinoline | 5.999 | C_14_H_13_NO_4_ | [M+H]^+^ | 260.09149 | 260.0915/245.0587/227.0911/201.0755 |
| 37 | 5-Phenyllevulinic acid | 6.062 | C_11_H_12_O_3_ | [M+ NH_4_]^+^ | 210.11224 | 210.1122/ |
| 77 | Hydroxyphenyllactate | 9.31 | C_11_H_8_O_4_ | [M+Na]^+^ | 205.04929 | 205.0493/145.0494 |
| 117 | (9*Z*)-5,8,11-Trihydroxyoctadec-9-enoic acid | 16.53 | C_18_H_34_O_5_ | [M+H]^+^ | 353.22943 | 353.2294/296.2301 |
| 85 | Perillyl alcohol | 10.432 | C_10_H_16_O | [M+H]^+^ | 153.12717 | 153.1272/135.1168 |
| 118 | Lauryldiethanolamine | 19.443 | C_16_H_35_NO_2_ | [M+H]^+^ | 274.27359 | 274.2740/256.2635 |
| 119 | Gelomulide N | 21.54 | C_24_H_32_O_7_ | [M-H_2_O+H]^+^ | 415.21091 | 415.2010/135.0805/119.0856 |

**Table S4.** Predicted absorption and distribution parameters of identified metabolites from *P. pyrifolia.*

| **No.** | **Compound** | **HIA (%)** | **Caco-2** | **PPB (%)** | **Bioavailability (%)** |
| --- | --- | --- | --- | --- | --- |
| 3 | 2-Hydroxy-3,4-dimethoxybenzoic acid | 94.3 | 0.571 | 59.1 | 56 |
| 4 | Gentiobiose | 12.3 | 0.005 | N/A | 17 |
| 6 | D-1-[(3-carboxypropyl)amino]-1-deoxyfructose | 14.6 | 0.015 | N/A | 55 |
| 7 | D-Sorbitol | 18.3 | 0.026 | N/A | 55 |
| 8 | Mycosporine serinol | 51.9 | 0.096 | N/A | 55 |
| 10 | Tyrosine | 57.2 | 0.042 | 5.0 | 55 |
| 11 | Methyl 3,4,5-trihydroxycyclohexene-1-carboxylate | 56.4 | 0.068 | 0.1 | 55 |
| 13 | Noradrenaline | 58.7 | 0.054 | 2.3 | 55 |
| 14 | Norleucine | 90.7 | 0.256 | 66.1 | 85 |
| 26 | Methoxyindoleacetic acid | 63.8 | 0.118 | 35.3 | 55 |
| 30 | [(2*S*,3*R*,4*S*,5*S*,6*R*)-3,4,5-trihydroxy-6-(hydroxymethyl)oxan-2-yl] (*E*)-3-phenylprop-2-enoate | 84.6 | 0.441 | 35.2 | 55 |
| 32 | Pyrogallol | 38.7 | 0.036 | 1.3 | 56 |
| 35 | N-acetylleucine | 96.5 | 0.763 | 99.2 | 55 |
| 36 | Pantothenic acid | 99.4 | 0.987 | 77.2 | 55 |
| 37 | Methyl asterrate | 90.5 | 0.690 | 52.9 | 85 |
| 77 | 4,7,8-Trimethoxyfuro[2,3-b]quinoline | 74.8 | 0.126 | 35.2 | 56 |
| 85 | 5-Phenyllevulinic acid | 95.8 | 0.955 | 45.0 | 55 |
| 117 | Hydroxyphenyllactate | 60.8 | 0.028 | 72.2 | 56 |
| 118 | (9*Z*)-5,8,11-Trihydroxyoctadec-9-enoic acid | 98.1 | 0.977 | 85.9 | 55 |
| 119 | Perillyl alcohol | 88.6 | 0.818 | 83.5 | 55 |

*HIA: Human intestinal absorption; Caco-2: Caco-2 cell permeability; BBB: Blood-brain barrier penetration; PPB: Plasma protein binding.*

**Table S5.** Predicted metabolism and toxicity parameters of identified metabolites from *P. pyrifolia.*

| **No.** | **Compound** | **HLM (%)** | **DILI** | **Ames Test** | **Overall Safety** |
| --- | --- | --- | --- | --- | --- |
| 3 | 2-Hydroxy-3,4-dimethoxybenzoic acid | 2.9 | Safe | Negative | Acceptable |
| 4 | Gentiobiose | 4.6 | Safe | Negative | Acceptable |
| 6 | D-1-[(3-carboxypropyl)amino]-1-deoxyfructose | 4.1 | Safe | Negative | Acceptable |
| 7 | D-Sorbitol | 4.0 | Safe | Negative | Acceptable |
| 8 | Mycosporine serinol | 9.3 | Safe | Negative | Acceptable |
| 10 | Tyrosine | 0.8 | Safe | Negative | Acceptable |
| 11 | Methyl 3,4,5-trihydroxycyclohexene-1-carboxylate | 4.5 | Safe | Negative | Acceptable |
| 13 | Noradrenaline | 1.7 | Safe | Negative | Acceptable |
| 14 | Norleucine | 2.7 | Safe | Negative | Acceptable |
| 26 | Methoxyindoleacetic acid | 11.4 | Risk | Negative | Caution |
| 30 | [(2*S*,3*R*,4*S*,5*S*,6*R*)-3,4,5-trihydroxy-6-(hydroxymethyl)oxan-2-yl] (*E*)-3-phenylprop-2-enoate | 8.4 | Risk | Positive | Caution |
| 32 | Pyrogallol | 4.1 | Safe | Negative | Acceptable |
| 35 | N-acetylleucine | 49.4 | Safe | Negative | Acceptable |
| 36 | Pantothenic acid | 29.5 | Risk | Positive | Caution |
| 37 | Methyl asterrate | 5.0 | Safe | Negative | Acceptable |
| 77 | 4,7,8-Trimethoxyfuro[2,3-b]quinoline | 3.3 | Safe | Negative | Acceptable |
| 85 | 5-Phenyllevulinic acid | 10.7 | Safe | Negative | Acceptable |
| 117 | Hydroxyphenyllactate | 2.6 | Safe | Negative | Acceptable |
| 118 | (9*Z*)-5,8,11-Trihydroxyoctadec-9-enoic acid | 67.2 | Risk | Positive | Caution |
| 119 | Perillyl alcohol | 36.6 | Risk | Positive | Caution |

*HLM: Human liver microsome stability (% metabolized); DILI: Drug-induced liver injury risk (Safe / Risk); Ames: Ames mutagenicity test result.*
