# Supplementary material for: Pear Peel–Enriched Residue Extract Attenuates Ethanol‐Induced Liver Injury Through Regulation of Antioxidant Signaling and Lipid Metabolism
Source: Adv Pharmacol Pharm Sci. 2026 Nov 4;2026:6147577. doi: 10.1155/adpp/6147577 (PMC13735535; doi:10.1155/adpp/6147577)
Supplement: Supplementary file 2 [file ADPP-2026-6147577-s002.docx]

**Figure S1. Antioxidant characterization of pear-derived extracts.** (A) Total phenolic content (TPC) of pear extract (PE) obtained from the whole fruit and pear peel-enriched residue extract (PPE) prepared from the peel-rich residue remaining after juice extraction. TPC was determined using the Folin–Ciocalteu method and expressed as mg gallic acid equivalents (GAE) per 100 g extract. (B) DPPH radical-scavenging activity of PE and PPE measured using the DPPH assay at different concentrations (50, 250, and 500 μg/mL). Ascorbic acid (AA, 100 μg/mL) was used as the reference antioxidant. Values represent the percentage inhibition of DPPH radicals.

**Figure S2.** Relative content of detected compounds from *P. pyrifolia* extract based on calculation of peak areas.

**Figure S3.** Interactions of ligands [ruby (3), deep purple (4), sand (6), limegreen (7), red (8), magenta (10), orange (11), hotpink (13), raspberry (14), forest (26), violet (30), salmon (32), tv-orange (36), wheat (85), skyblue (118), limon (119)] with 1HDZ protein.

**Figure S4.** Interactions of ligands [ruby (3), deep purple (4), sand (6), limegreen (7), red (8), magenta (10), orange (11), hotpink (13), raspberry (14), forest (26), violet (30), salmon (32), tv-orange (36), wheat (85), skyblue (118), limon (119)] with 1XKT protein.

**Figure S5.** Interactions of ligands [ruby (3), deep purple (4), sand (6), green (7), red (8), magenta (10), limegreen (11), hotpink (13), violet (14), forest (26), wheat (30), magenta (32), lime (36), warmpink (85), skyblue (118), limon (119)] with 3GLU protein.

**Figure S6.** interactions of ligands [ruby (3), chocolate (4), sand (6), green (7), deeppurple (8), magenta (10), orange (11), red (13), violet (14), brown (26), wheat (30), hotpink (32), forest (36), raspberry (85), marine (118), limon (119)] with 4H80 protein.
